# Toward the standardization of big datasets of urine output for AKI analysis: a multicenter validation study

**DOI:** 10.1038/s41598-025-95535-4

**Published:** 2025-06-06

**Authors:** Ariel Avraham Hasidim, Matthew Adam Klein, Itamar Ben Shitrit, Sharon Einav, Karny Ilan, Lior Fuchs

**Affiliations:** 1 Department of Epidemiology, Biostatistics and Community Health, Faculty of Health Sciences, Ben-Gurion University, Beer-Sheva, Israel; 2 Department of Pediatrics A, Schneider Children’s Medical Center of Israel, Petah Tikva, Israel; 3 Sackler Faculty of Medicine, Tel Aviv University, Tel Aviv, Israel; 4 Department of Internal Medicine, Mayo Clinic, Jacksonville, FL USA; 5 Joyce and Irving Goldman Medical School, Faculty of Health Sciences, Ben-Gurion University of the Negev, Beer-Sheva, Israel; 6 Clinical Research Center, Faculty of Health Sciences, Soroka University Medical Center, Ben-Gurion University of the Negev, Beer-Sheva, Israel; 7 Maccabi Healthcare Services and Hebrew University Faculty of Medicine, Jerusalem, Israel; 8 General Surgery Department, Sheba Medical Center, Ramat-Gan, Israel; 9 Medical Intensive Care Unit and Clinical Research Center, Faculty of Health Sciences, Soroka University Medical Center, Ben-Gurion University of the Negev, Beer-Sheva, Israel

**Keywords:** Acute kidney injury, Data processing

## Abstract

Acute kidney injury (AKI) is a prevalent condition in ICU patients. However, inconsistencies in urine charting and guideline interpretations hinder accurate diagnosis and research. This study aimed to derive and validate a standardization for the processing of big urine output datasets to improve consistency in AKI diagnosis and staging. Using a derivation cohort from 14 ICUs at Beth Israel Deaconess Medical Center (2008–2019) and a validation cohort from an academic center in Amsterdam (2003–2016), we developed and validated an algorithm for computing hourly urine output rates and identifying oliguric AKI across its definitions. Peak AKI stages computed using the method were significantly associated with all clinical outcomes, including severity scores, serum creatinine levels, ICU and hospital lengths of stay, renal replacement therapy requirements, and hospital mortality (all p < 0.001). Adjusted 30-day mortality odds ratios for AKI stages 1–3 were 1.58, 2.93, and 5.24 in the derivation cohort and 2.91, 5.16, and 13.59 in the validation cohort (all p < 0.001). Tested on over 85,000 multinational ICU admissions, this approach demonstrated robust performance and consistent results across diverse settings; it has the potential to enhance clinical practice through e-alerts and support future AKI and fluid balance research, including ML model training and inference. Supported by open-source code, the proposed method advances the standardization of AKI diagnostic criteria and can be applied to other EHR-based databases.

## Introduction

*Acute kidney injury* (AKI) affects 30–50% of critically ill patients, with severe cases requiring *renal replacement therapy* (RRT), which is associated with mortality rates up to 50%^1–3^. These outcomes may be mitigated with timely treatment such as fluid adjustment or earlier RRT initiation^4–6^, as *urine output* (UO) monitoring in AKI patients has been associated with improved survival rates^7,8^. Using only *serum creatinine* (sCr) without UO for diagnosing AKI may reduce sensitivity, delay detection, and underestimate mortality^3,9–12^. Despite relying on KDIGO guidelines^13^, many studies and e-alerts exclude UO altogether from AKI definitions, mostly due to infeasibility or unavailability^9,11,14–16^.

The KDIGO guidelines define two sets of criteria for diagnosing and staging AKI, using UO or sCr^13^. Two interpretations for the *UO criteria* (KDIGO-UO) are customary^11,17–19^: (1) where the *average ml/kg/hr over 6, 12, and 24-h windows meet the threshold* (UO^mean^)^20–22^; (2) where *UO meets KDIGO’s threshold in each consecutive hour* (UO^cons^)^23–28^. The latter often requires handling missing hourly data, as each hour should be addressed individually^18^. A comparison of these approaches has shown differences in diagnosis rates and associated outcomes^11,17–19^.

Retrospective UO analyses are limited by inconsistent time intervals, simultaneous charting, and various collection methods other than urethral catheterization (e.g., spontaneous voiding, nephrostomies, etc.). Current approaches for UO data handling include total interval summation (e.g., for 12- or 24 h)^11,29,30^, including only cases with complete hourly measurements using Foley catheter^18,31^, and hourly imputation techniques such as dividing cumulative volume by the number of missing hours^17,20,21,32–34^, using linear interpolation^11^, or using *machine learning* (ML)-based imputation models^35–37^.

Moreover, some studies do not explicitly state the UO data handling approach^22,38^ or KDIGO-UO interpretation^31–34,38^, which may additionally limit reproducibility.

Current approaches for handling UO data offer either low temporal resolution or rely on various imputation techniques. Imputation of missing measurements by dividing the volume over the missing hours suits studies that include only urethral catheterization cases but is ineffective for multiple sources or simultaneous measurements. ML-based imputation acts as a “black box” with variable effectiveness depending on the dataset and algorithm used; It often lacks reproducibility and interpretability and carries a high risk of bias^37^.

The lack of standardization in handling UO data and the various interpretations of KDIGO-UO guidelines limit the ability to make consistent comparisons and draw general conclusions. This proof-of-concept study aimed to establish a straightforward method for standardizing the computation of hourly UO for the study of oliguric AKI using real-life charting data. We also sought to validate this method on a separate dataset. We hypothesized that a generalizable approach could be established despite existing charting practices and that this method would consistently enable the detection of AKI for its various interpretations. We hereby present the developed method.

## Materials and methods

This retrospective analysis was conducted using real-time data from two publicly available databases. The findings are reported according to the *REporting of studies Conducted using Observational Routinely-collected Data* (RECORD) statement^39^.

The data used for method derivation were taken from the Medical Information Mart for Intensive Care IV 2.2 database (MIMICdb)^40–44^. The data used for method validation was taken from the Amsterdam University Medical Center database (AUMCdb)^45^. The datasets contain data on patients admitted to ICUs at the Beth Israel Deaconess Medical Center (BIDMC) between 2008 and 2019 and the academic medical center in Amsterdam between 2003 and 2016. The MIMICdb was selected for derivation since it comprises a larger cohort and a more comprehensive set of potential confounders. Intensivists and public health specialists conducted all data analyses at the Ben Gurion University and Soroka Medical Center.

All methods were performed in accordance with the relevant guidelines and regulations. Institutional review boards at BIDMC and MIT approved the MIMIC-IV project. Due to the study’s retrospective nature, informed consent was waived, and the medical information was deidentified according to the health insurance portability and accountability act (HIPAA) mandated standards^40^. The privacy audit of AmsterdamUMCdb determined the data to be effectively anonymous under the European general data protection regulation (GDPR), waiving the need for informed consent^45,46^.

Patients admitted to an adult ICU with documented UO measurements were considered eligible for inclusion in both method derivation and validation. Eligibility for analysis of AKI also required that the KDIGO-UO staging for the first day of admission could be computed (i.e., data were available on admission weight and at least six consecutive hours of valid hourly-adjusted UO). To avoid bias from repeated measurements and multiple instances per survival outcome, AKI analysis included only each patient’s first ICU admission.

For the derivation cohort, we used MIMICdb ICU admissions, classified by the specific treating medical service^47^. We included data from 14 types of ICUs: non-surgical (medical, cardiac, neurologic, orthopedic) and surgical (general, thoracic, cardiac, trauma, neurologic, orthopedic, ENT, plastic, vascular, genitourinary). Data from obstetric, gynecologic, and dental ICUs were excluded due to limited documentation of UO.

For the validation cohort, we used data from all adult ICU stays in the AUMCdb, excluding medium care units^45^.

### Outcomes

The primary study outcome was using the MIMICdb to derive a computation for an hourly UO adjustment method as a generalized code. The secondary study outcomes were (1) to demonstrate that oliguric AKI events can be identified in the original database using the method (AKI event validity confirmed by comparing the rates of AKI with indirect indicators of AKI such as survival) and (2) to demonstrate that the derived method and its code may be similarly used in a second ICU database (external validation).

### Data sources

The data extracted from the MIMICdb included patient demographics (age, gender, weight, ethnicity), comorbidities (chronic kidney disease [CKD], diabetes mellitus [DM], and charlson comorbidity index [CCI]), severity at the time of ICU admission (SOFA and SAPS-II scores), sCr values during ICU stay, use of RRT, ICU *length of stay* (LOS) and hospital LOS and mortality. Most of these variables were also available in the AUMCdb. Both datasets included documentation of urine volume, charting time, and collection method (catheter, voiding, etc.). The durations of UO collection were not recorded in either database.

The data from the two databases were captured in real time by the treating staff members and hospital systems. Raw variables were obtained directly from the original tables, including age, gender, weight, ethnicity, serum creatinine levels, and length of stay. CKD was detected using ICD-9: 585.x and ICD-10: N18.x codes. DM was detected using ICD-9: ICD-9: 2500-3, 2508-9 and ICD-10: E100-1, E106, E108-11, E116, E118-21, E126, E128-31, E136, E138-41, E146, E148-9 codes. The variables KDIGO-Cr stageing, SOFA score, SAPS-II score, RRT, and CCI were extracted from the derived data in the official MIMICdb’s code repository^48–52^.

A negligible number of admissions with UO records originating from “urethral stents” were excluded due to uncertainty and the possibility of post-renal obstruction. Admissions with “GU irrigation” UO records were excluded only from the MIMICdb dataset due to confounding by input^53^. Admissions with “urinary leak” UO records were excluded from the AUMCdb dataset due to potential impacts on record reliability.

Age and weight were stored in the AUMCdb as 10-unit range groups. Therefore, these values were handled as group averages. Extreme groups were assigned tail values plus 5 units (e.g., age “80+ ” was regarded as 85).

### Computation of hourly-adjusted UO

Raw UO data was processed to derive the hourly-adjusted UO as follows: The duration of collection of each documented volume was calculated based on the time interval from the previous urine measurement, as suggested by the MIMIC website^54^. Time intervals were calculated in each physiological compartment separately (i.e., urinary bladder, L/R nephrostomies, Ileoconduit). Next, UO rates were calculated for each duration. Finally, for a given calendar hour in the ICU, hourly-adjusted UO was determined by summing the overlapping UO rates of each compartment. An hourly-adjusted value was computed only when a UO rate was present for most of the calendar hour (Supplemental Methods 1).

### Classification of AKI and staging

Hourly KDIGO-UO staging was defined in two ways: (1) UO^mean^, calculated as the running average UO (UO/kg/hr) over the last 6-, 12-, and 24 h windows; and (2) UO^cons^, determined by comparing the highest hourly UO/kg within each window with the corresponding KDIGO threshold. A complete dataset of consecutive hourly-adjusted UO measurements for the entire window was required for stage calculation (i.e., the first KDIGO staging could be calculated after completing the sixth hour of data).

### Addressing bias

To mitigate selection bias, data from all eligible ICU admissions meeting inclusion and exclusion criteria were incorporated. We reported the number of unplanned exclusions related to missingness to address bias due to missing data. Weight at admission is a key element in oliguric-AKI diagnosis and staging; A sensitivity analysis using multiple imputations was planned but deemed unnecessary, as missing values did not exceed 5%^55^. We analyzed temporal trends in UO charting practices to account for them (Supplemental Appendix 1). UO charting times were used to mark the durations of the UO collection after confirming the absence of duplicate or simultaneous entries (Supplemental Appendix 2). A sensitivity analysis was conducted to study the potential effect of different thresholds for excluding outliers of prolonged durations of collection; No significant differences in the diagnosed population were found (Supplemental Table 1). We compared UO^mean^ and UO^cons^; UO^mean^ was calculated twice, before and after the hourly adjustment (Supplemental Appendix 3); UO^cons^ was preferred for the rest of the study. External validation was performed to reduce the risk of Type II errors inherent in models derived from existing data.

The minimal detectable differences in RRT, mortality, and AKI incidences were calculated for several sample sizes for a power of 0.8 and p-value of 0.05 (Supplemental Methods 2).

### Statistical analyses

Charting practices were studied, including the range of values for the duration of UO collection. Illogical UO measurements with volumes below 0 or above 5000 ml were excluded. Admission weights > 300 kg or < 25 kg were treated as artifacts, and the ICU stays were excluded^56^.

Categorical variables were presented as frequencies and percentages. Continuous variables were reported using either mean and standard deviation or median and interquartile range, depending on the variable distribution. Averaged UO rates were presented after weighting based on the collection durations. Categorical variables were compared using a Chi-squared test or Fisher exact test. Means were compared using the t-test or ANOVA, and medians were compared using Wilcoxon or Kruskal–Wallis rank sum tests.

AKI rates at the first days of admission and their associated outcomes were described for the first 72 h after the first UO record. Admissions were stratified by maximal KDIGO-UO staging in the first three days. For prevalence, the rate of AKI at admission was described.

For survival analysis, follow-up times were calculated from admission time. Kaplan–Meier (KM) plots with log-rank test, univariate and multivariate logistic regression were used to assess 30-day survival, unadjusted and adjusted Odds Ratios (OR) for mortality. Adjusted ORs were presented as marginal effects after accounting for age, weight, gender, and whether the diagnosis was made upon admission. The covariate *diagnosis at admission* was used to address the uncertainty associated with KDIGO-UO labeling for events that may have started before ICU admission. Each covariate and its interactions were included as predictors.

For the secondary outcome of demonstrating that the derived method may be similarly used in a second ICU database (external validation), the entire procedure was replicated on the AUMCdb. Both datasets followed standard UO charting, including volume, source, and timestamp. The only differences were source labeling language and the exclusive presence of “Straight Cath” and “Condom Cath” in MIMICdb. After translation, the code was applied to AUMCdb without modification.

The data for this study were extracted using Google Cloud Platform’s “BigQuery” service with Standard SQL dialect. RStudio (4.3.1) and R packages were used for all data analyses. All code is open-sourced and publicly available, as described below.

## Results

**Fig. 1:**
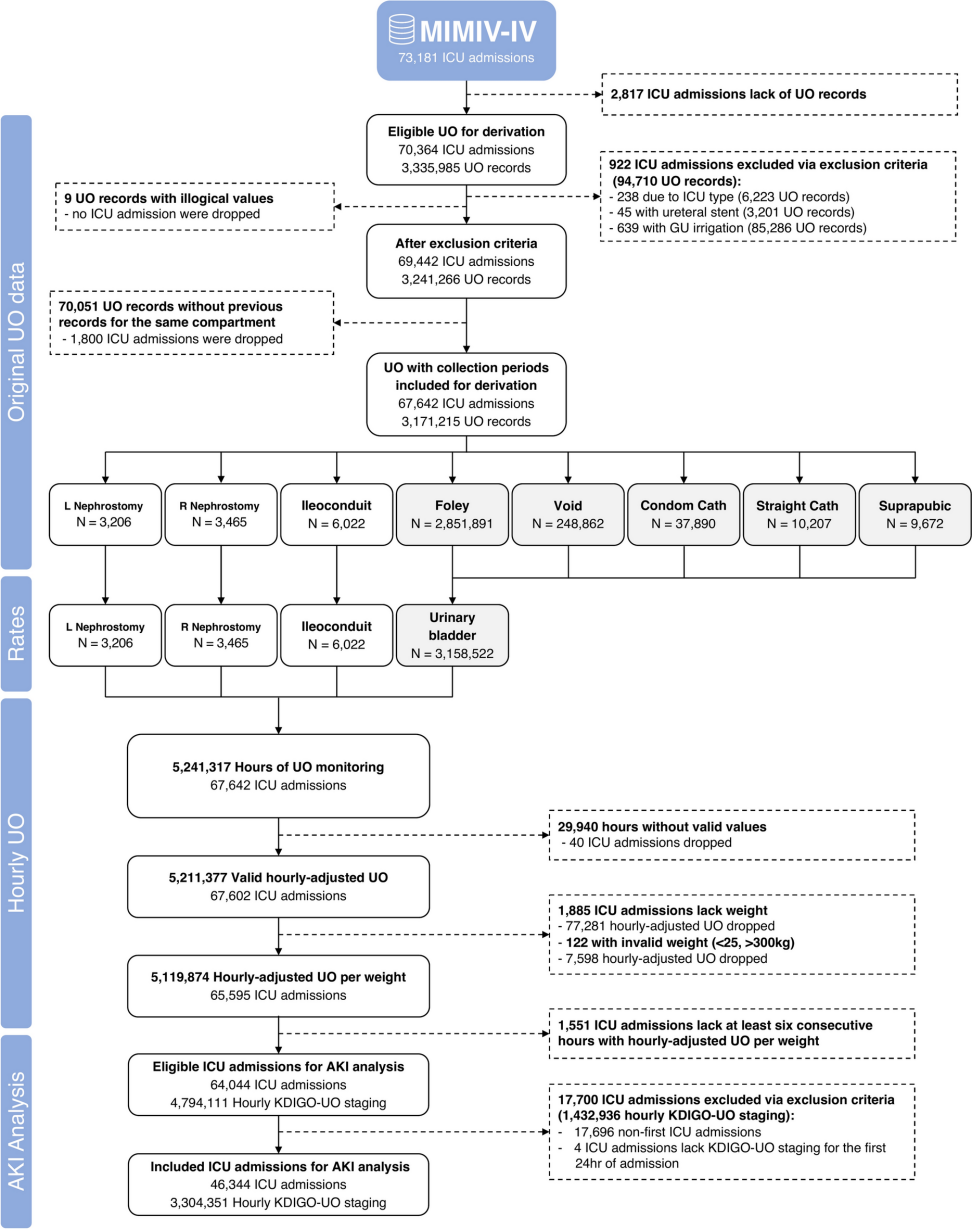
MIMICdb data inclusion flow for computing hourly-adjusted UO and AKI analysis. UO sources were categorized into four physiologic compartments of urine accumulation. The first measured volume in each compartment was treated as residual volume from before admission and was dropped. UO rates were calculated by identifying time intervals from the last known record in the same compartment as the durations of collection. Valid hourly-adjusted UO was computed only when the UO rate was present for most of the calendar hour. *GU* genitourinary, *ICU* intensive care unit, *L* left, *R* right, *UO* urine output.

The MIMICdb yielded 3,335,985 eligible UO records before exclusions across 49,950 patients, 64,110 hospitalizations, and 70,364 ICU admissions. The inclusion–exclusion process is presented in (Fig. 1). Twelve UO collection methods were identified and classified into four separate compartments, with Foley and urinary bladder being the most frequent (Supplemental Fig. 1). After applying exclusion criteria, 96.1% of eligible admissions (67,642/70,364) and 95.1% of eligible urine measurements (3,171,215/3,335,985) were included, representing 218,388 days of UO monitoring in the ICU. The average UO durations of collection and measured volume in the MIMICdb were 99 (SD, 176) minutes and 135 (SD, 144) ml.

Demographics and clinical characteristics for ICU admission are presented in (Table 1). 29,686 (44%) were females, their respective average age and weight at admission were 65 (SD, 17) kg and 81 (SD, 34) years (see Supplemental Appendix 4). Their respective median CCI, SOFA and SAPS-II scores at ICU admission were 5 (IQR, 3–7), 4 (IQR, 2–6), and 33 (IQR, 25–32).

**Table 1:** Patient demographics for ICU admission in MIMICdb and AUMCdb.

Characteristic	N	MIMICdb N = 67,642^*1*^	**N**	AUMCdb N = 17,736^*1*^	p-value^*2*^
Age at hospital admission, years	67,642	65 (17)	17,736	63 (15)	< 0.001
Weight at ICU admission, kg	65,751	81 (34)	17,143	81 (15)	< 0.001
Gender	67,642		17,320		< 0.001
F		29,686 (44%)		5,705 (33%)	
M		37,956 (56%)		11,615 (67%)	
Ethnicity	60,412		0		
African American		6,836 (11%)			
Asian		1,956 (3.2%)			
Caucasian		46,323 (77%)			
Hispanic		2,484 (4.1%)			
Other		2,813 (4.7%)			
SAPS-II at ICU admission	67,161	33 (25, 42)	0		
SOFA score at ICU admission	67,642	4 (2, 6)	0		
CCI score	67,642	5 (3, 7)	0		
CKD, stage 1–4	67,624	13,162 (19.5%)	0		
Diabetes mellitus	67,624	15,896 (23.5%)	0		
First sCr in ICU, mg/dL	67,287	1.35 (1.37)	17,677	1.21 (1.06)	< 0.001
Peak sCr at first days, mg/dL	67,263	1.52 (1.55)	16,962	1.34 (1.16)	< 0.001
ICU discharge sCr, mg/dL	67,287	1.25 (1.21)	17,677	1.10 (0.84)	< 0.001
Peak KDIGO-Cr at first days	66,255		0		
0		48,817 (74%)			
1		12,222 (18%)			
2		2,764 (4.2%)			
3		2,452 (3.7%)			
Time in hospital, days	67,642	7.0 (4.0, 13.0)	0		
Time in ICU, days	67,642	2.0 (1.1, 3.8)	17,736	1.3 (0.9, 4.7)	< 0.001
Renal replacement therapy	67,642	4,009 (5.93%)	17,736	1,047 (5.90%)	> 0.9
Hospital mortality	67,642	7,198 (10.6%)	17,736	2,010 (11.3%)	0.008

*CCI* charlson comorbidity index, *CKD Stage 1–4* chronic kidney disease excluding end-stage-renal-disease, *sCr* serum creatinine, *DM* diabetes mellitus, *ICU* intensive care unit, *SOFA* sequential organ failure assessment.

^*1*^Mean (SD); n (%); Median (Q1, Q3).

^*2*^Welch two sample t-test; Pearson’s Chi-squared test; Fisher’s exact test; Wilcoxon rank sum test.

### Primary outcome: computing hourly-adjusted UO (method derivation)

**Fig. 2:**
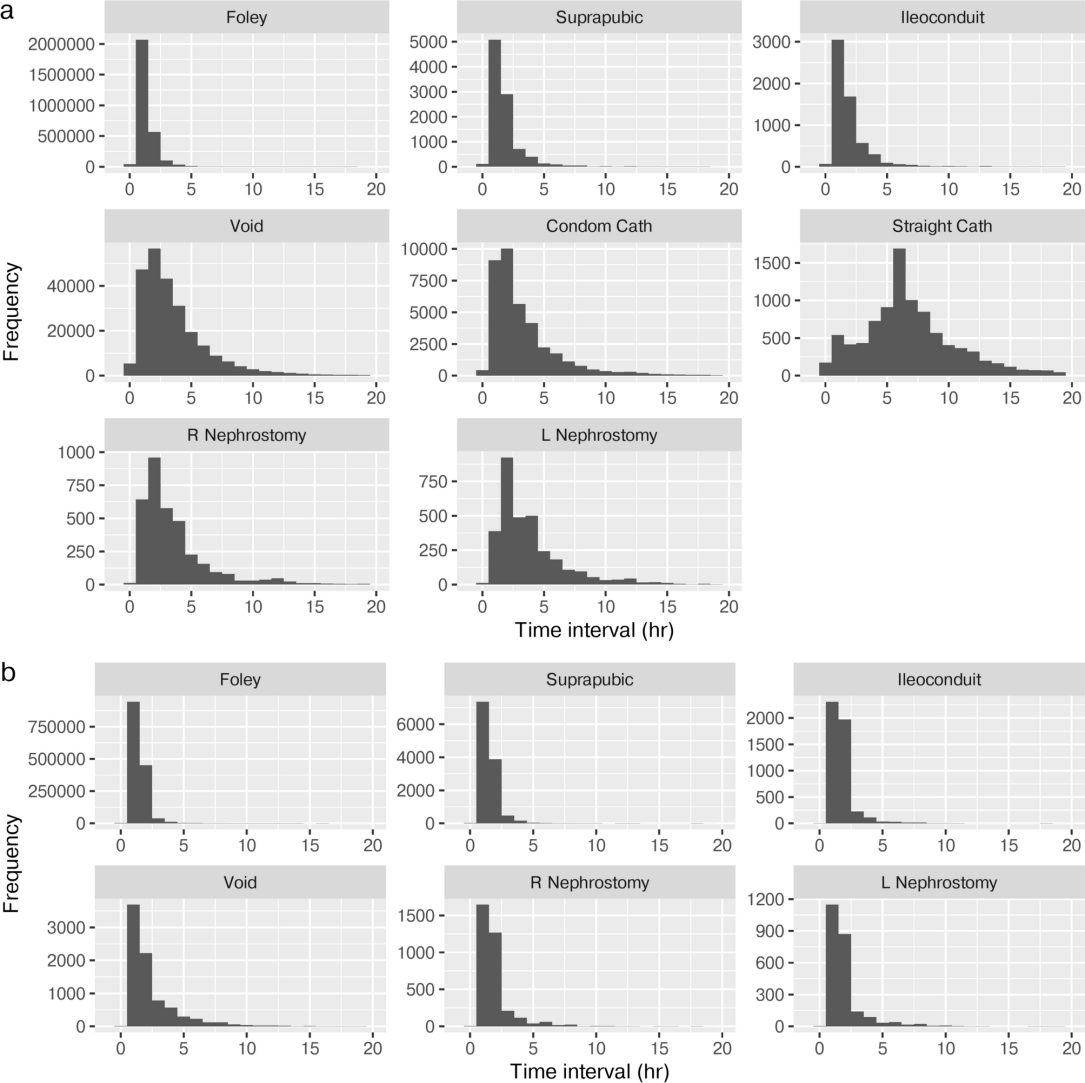
Durations of collection (hours). Durations of collection for all volume measurements histograms. Panel (**a**) plotted for MIMICdb; Panel (**b**) plotted for AUMCdb. For the N count for each source, see inclusion flow figures. *L* left, *R* right.

The durations of collection for Foley, suprapubic, and ileal conduit catheters were short (mode and median 60 min). Durations were intermediate for voiding and condom catheters with a right-skewed distribution (mode 120 both, median 180 and 131 min, respectively). Straight catheters had long durations of collection (mode 360, median 409 min). The durations of collection with left and right nephrostomies were similar and had a distinct distribution (mode 120, median 180 min) (Fig. 2 and Supplemental Table 2).

The averaged volumes were proportional to the durations of collection. The proportion of “zero volume” records (i.e., no UO) ranged between 1–2.5% across all measurement sources (Supplemental Fig. 2) and had longer durations in average (Supplemental Table 3). At urine production rates above 20 ml/hr, there was a linear association between the smoothed conditional means of the durations of collection and volumes. At production rates below 20 ml/hr, durations were more prolonged (validated in quantile analysis, Supplemental Appendix 5).

The weighted average rate of UO for both kidneys (represented by Foley catheter) was 85.1 ml/hr. The weighted average rates for a single kidney (represented by R/L nephrostomies) were 39.9 and 42.1 ml/hr (Table 2).

**Table 2:** Comparison of urine output rates by collection source.

Source	MIMICdb	AUMCdb
Foley	85.1	91.2
Suprapubic	66.0	83.3
Ileoconduit	68.8	60.5
Void	77.3	81.1
Condom cath	66.3	–
Straight cath	40.8	–
R nephrostomy	42.1	30.7
L nephrostomy	39.9	31.8

The table presents the weighted mean hourly urine output rate (ml/hr) for all urine output sources, with weights based on the collection period of each rate.

**Fig. 3:**
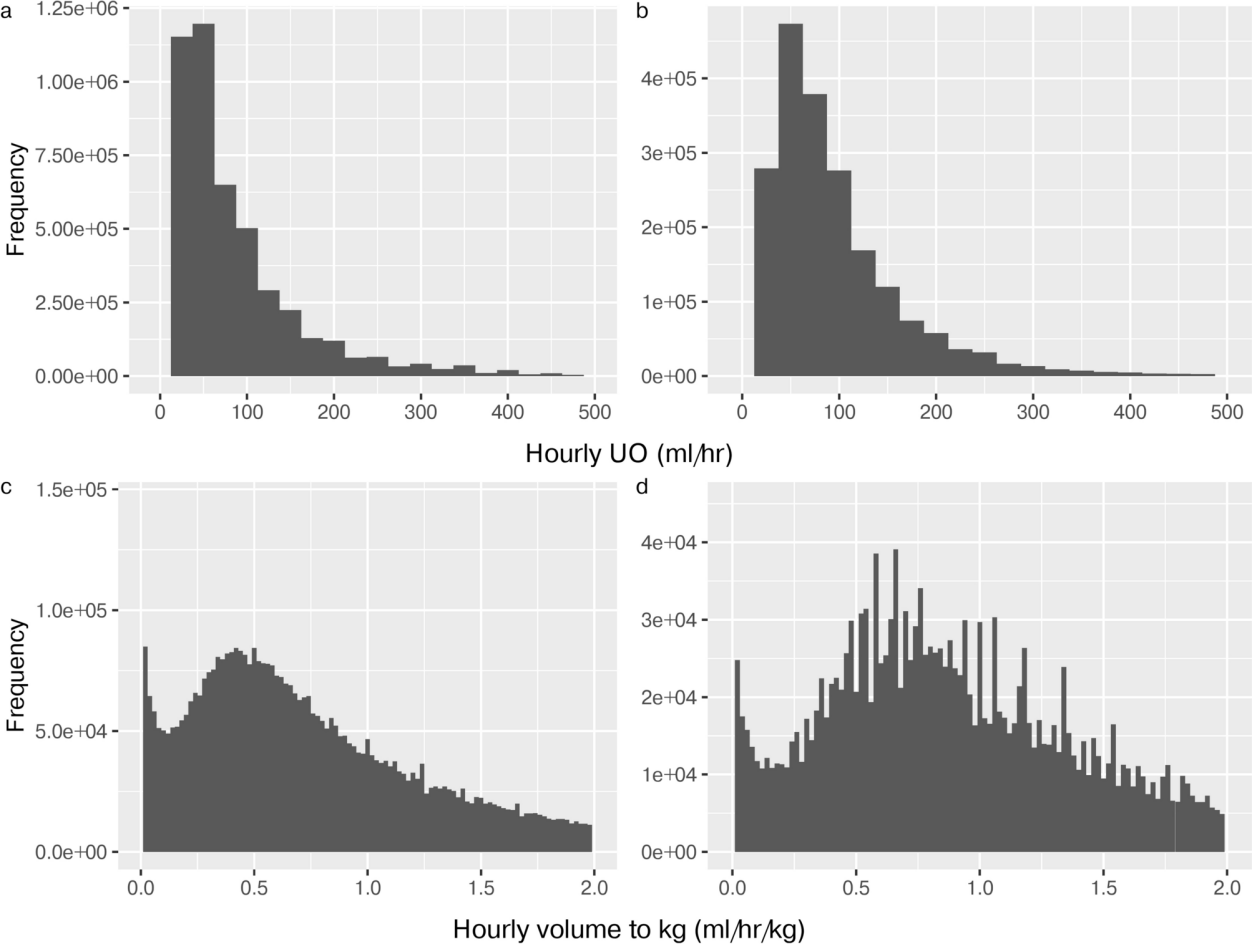
Histograms for hourly-adjusted UO. We can universally see a bimodal, smooth, and homogenous distribution with a positive skew and no significant outliers, suggesting natural variability in the study population without extreme values, data entry errors, or distinct sub-populations. In panel d we can see a similar pattern, but without smoothing due to weight being grouped in 10 kg intervals in the AUMCdb. Panel (**a**) shows the hourly-adjusted UO plotted for MIMICdb (N = 5,211,377); Panel (**b**) shows the hourly-adjusted UO plotted for AUMCdb (N = 2,140,972); Panel (**c**) shows the hourly-adjusted UO per kg plotted for MIMICdb (N = 5,119,874); Panel (**d**) shows the hourly-adjusted UO per kg plotted for AUMCdb (N = 2,063,720). *L* left, *R* right, *UO* urine output.

A valid hourly-adjusted UO value was available for 99.4% of the included hours. Descriptive statistics showed bimodal distribution and characteristics consistent with valid observations (Fig. 3).

### Secondary outcome: identification of acute kidney injury (derivation cohort)

**Fig. 4:**
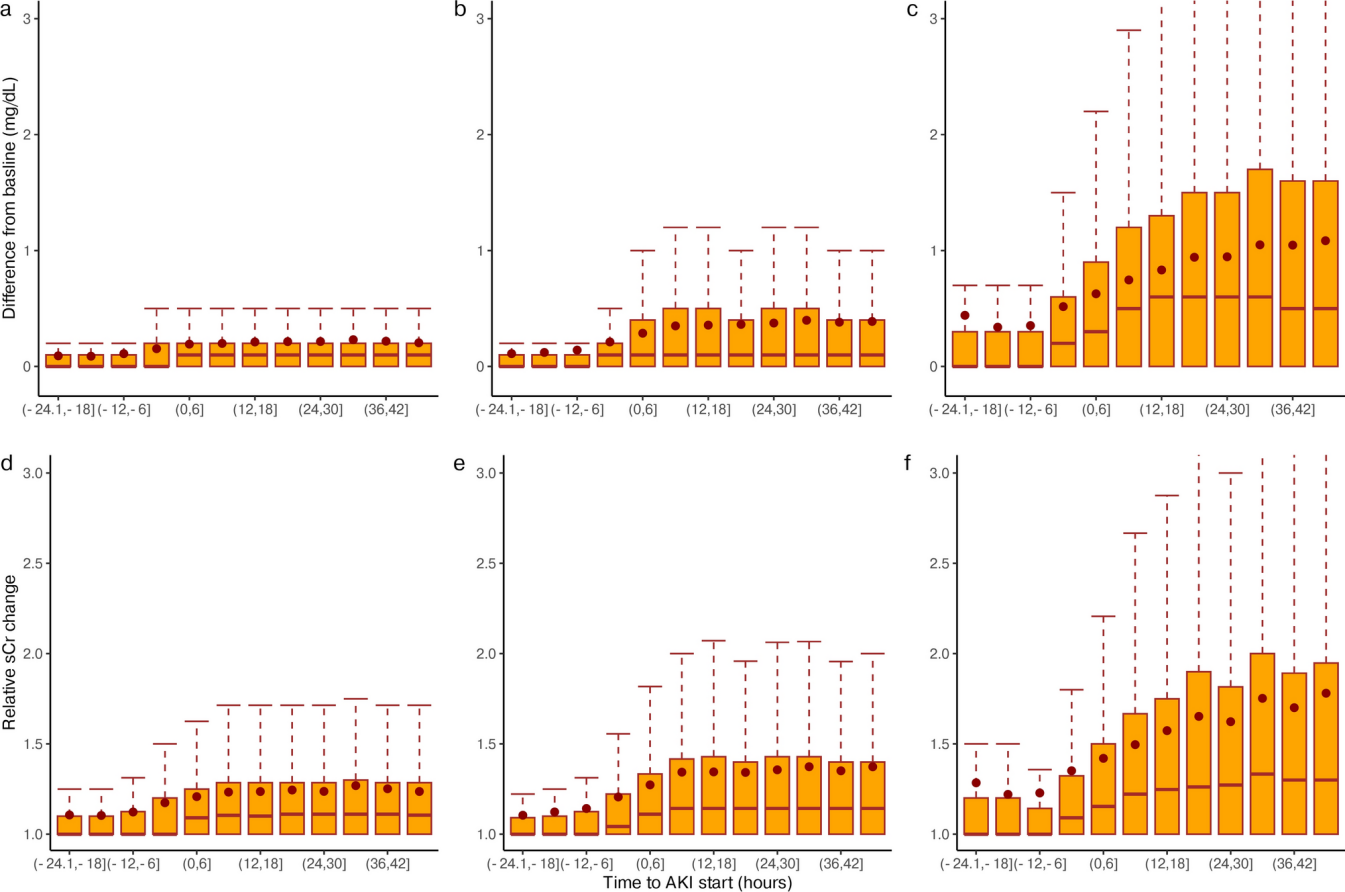
Change in serum creatinine levels. The figure presents a two-part plot depicting box plots and mean change in serum creatinine from the lowest value in the 7 days preceding each measurement. Panels (**a**–**c**) shows the absolute change in mg/dL for maximum KDIGO-UO staging 1–3, while panels (**d**–**f**) shows the relative change for the same maximum staging, which is unitless. The x-axis represents the time to AKI onset in hours, ranging from 24 h before the event to 48 h after, in 6-h intervals.

Among 67,642 included ICU admissions, 64,044 met the minimal requirements for calculating KDIGO-UO stage. After excluding repeat ICU admissions, 46,344 admissions were included in the AKI analysis (Fig. 1). A total of 48.3% of ICU admissions were diagnosed with AKI in the first 3 days of admission by UO^cons^ (22,372/46,344), as shown in (Table 3). The prevalence at admission was 13.8% (6,399/46,344). The comparison of maximal KDIGO-UO staging showed a consistent association between increased staging and sCr levels (Fig. 4), severity scores, KDIGO-Cr staging, and clinical outcomes (all with p < 0.001) (Table 4).

**Table 3:** Incidence of oliguric-AKI on the first days at ICU.

Characteristic	MIMICdb N = 46,344^*1*^	AUMCdb N = 14,923^*1*^	p-value^*2*^
Oliguric-AKI on the first days	22,372 (48.3%)	4,688 (31.4%)	< 0.001
Maximum KDIGO staging			< 0.001
1	11,262 (50.3%)	2,456 (52.4%)	
2	8,991 (40.2%)	1,589 (33.9%)	
3	2,119 (9.47%)	643 (13.7%)	
Prevalence at admission	6,388 (13.8%)	1,024 (6.86%)	< 0.001

^*1*^n (%).

^*2*^Pearson’s Chi-squared test.

**Table 4:** ICU admission characteristics by maximal KDIGO-UO stage.

Characteristic	MIMICdb					AUMCdb				
	No AKI N = 23,972^*1*^	Stage 1 N = 11,262^*1*^	Stage 2 N = 8,991^*1*^	Stage 3 N = 2,119^*1*^	p-value^*2*^	No AKI N = 10,235^*1*^	Stage 1 N = 2,456^*1*^	Stage 2 N = 1,589^*1*^	Stage 3 N = 643^*1*^	p-value^*2*^
Age at hospital admission, years	62 (18)	67 (16)	68 (16)	66 (16)	< 0.001	62 (15)	65 (15)	68 (14)	66 (15)	< 0.001
Weight at ICU admission, kg	77 (20)	84 (23)	89 (27)	88 (28)	< 0.001	79 (14)	83 (15)	85 (15)	83 (16)	< 0.001
Gender					< 0.001					< 0.001
F	10,590 (44%)	4,710 (42%)	3,987 (44%)	963 (45%)		3,064 (31%)	879 (36%)	544 (35%)	231 (37%)	
M	13,382 (56%)	6,552 (58%)	5,004 (56%)	1,156 (55%)		6,933 (69%)	1,540 (64%)	1,028 (65%)	400 (63%)	
Ethnicity					< 0.001					
African American	2,051 (9.8%)	910 (9.3%)	840 (11%)	244 (14%)						
Asian	895 (4.3%)	232 (2.4%)	146 (1.9%)	59 (3.4%)						
Caucasian	16,023 (76%)	7,795 (80%)	6,228 (80%)	1,268 (73%)						
Hispanic	935 (4.5%)	325 (3.3%)	243 (3.1%)	67 (3.9%)						
Other	1,053 (5.0%)	471 (4.8%)	336 (4.3%)	95 (5.5%)						
CCI score	4 (2, 6)	5 (3, 7)	5 (3, 7)	6 (4, 8)	< 0.001					
CKD, stage 1–4	3,101 (13%)	1,802 (16%)	1,879 (21%)	799 (38%)	< 0.001					
Diabetes mellitus	4,933 (21%)	2,662 (24%)	2,201 (24%)	508 (24%)	< 0.001					
SOFA score at ICU admission	3 (1, 5)	4 (2, 6)	4 (2, 7)	8 (4, 12)	< 0.001					
SAPS-II at ICU admission	30 (22, 38)	33 (26, 42)	37 (29, 47)	49 (37, 60)	< 0.001					
APS-III at ICU admission	34 (26, 44)	38 (29, 50)	43 (33, 58)	64 (45, 83)	< 0.001					
First sCr in ICU, mg/dL	1.14 (1.04)	1.19 (1.02)	1.42 (1.51)	2.87 (2.81)	< 0.001	1.07 (0.65)	1.19 (0.93)	1.44 (1.50)	2.40 (2.42)	< 0.001
Peak sCr at first days, mg/dL	1.21 (1.07)	1.33 (1.10)	1.70 (1.70)	3.79 (3.07)	< 0.001	1.06 (0.64)	1.29 (0.94)	1.92 (1.59)	3.74 (1.98)	< 0.001
ICU discharge sCr, mg/dL	1.00 (0.79)	1.12 (0.95)	1.40 (1.38)	2.92 (2.46)	< 0.001	0.95 (0.50)	1.07 (0.81)	1.41 (1.20)	2.30 (1.50)	< 0.001
Peak KDIGO-Cr at first days					< 0.001					
0	20,132 (86%)	8,387 (75%)	5,646 (63%)	711 (34%)						
1	2708 (12%)	2,166 (19%)	2,247 (25%)	639 (30%)						
2	440 (1.9%)	369 (3.3%)	569 (6.4%)	204 (9.7%)						
3	175 (0.7%)	198 (1.8%)	454 (5.1%)	544 (26%)						
Time in hospital, days	6.0 (3.0, 10.0)	7.0 (4.0, 12.0)	8.0 (5.0, 13.0)	10.0 (5.0, 18.0)	< 0.001					
Time in ICU, days	1.4 (1.0, 2.5)	2.2 (1.3, 4.0)	2.9 (1.8, 5.1)	4.4 (2.8, 8.2)	< 0.001	1.0 (0.9, 2.0)	2.5 (1.0, 6.9)	4.7 (2.1, 11.3)	7.2 (3.0, 18.2)	< 0.001
Renal replacement therapy	281 (1.2%)	257 (2.3%)	604 (6.7%)	970 (46%)	< 0.001	44 (0.4%)	61 (2.5%)	213 (13%)	483 (75%)	< 0.001
Hospital mortality	1,163 (4.9%)	1,005 (8.9%)	1,440 (16%)	671 (32%)	< 0.001	424 (4.1%)	310 (13%)	389 (24%)	308 (48%)	< 0.001

*CCI* charlson comorbidity index, *CKD Stage 1–4* chronic kidney disease excluding end-stage-renal-disease, *sCr* serum creatinine, *DM* diabetes mellitus, *ICU* intensive care unit, *SOFA* sequential organ failure assessment.

^*1*^Mean (SD); n (%); Median (Q1, Q3).

^*2*^One-way analysis of means; Pearson’s Chi-squared test; Kruskal–Wallis rank sum test.

**Fig. 5:**
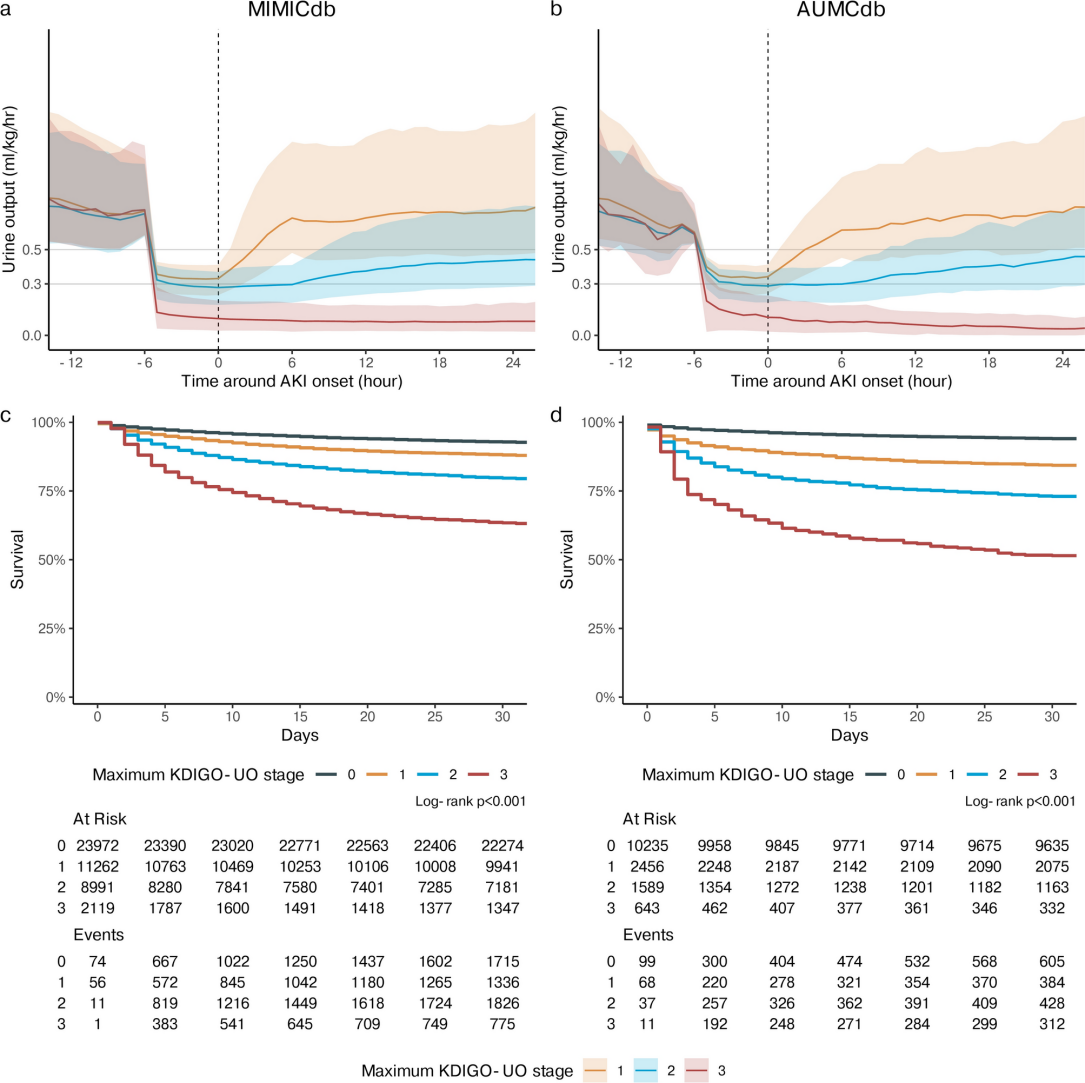
Hourly urine output and 30-day survival analysis. The top figures display the median and inter-quantile ranges for hourly UO per kg, along with the cutoff values for staging based on the KDIGO-UO guidelines. The bottom figures display KM plots for 30-day survival. Panel (**a)** shows the hourly urine output rate for the MIMICdb. Panel (**b)** shows the hourly urine output rate for the AUMCdb. Panel (**c)** shows survival for the MIMICdb. Panel (**d)** shows survival for the AUMCdb.

There is a significant difference between all KDIGO-UO stages for 30-day survival analysis (Log-Rank p < 0.001 for each between-group comparison) (Fig. 5) unadjusted and adjusted morality ORs (Table 5).

**Table 5:** Unadjusted OR for 30-day mortality by maximal KDIGO-UO.

	Patients, No. (%)	Mortality, No. (%)	Unadjusted OR		Adjusted Model^*1*^	
			OR (95% CI)	P value	OR (95% CI)	P value
MIMICdb						
No AKI	23,972 (0.52)	1,715 (0.07)	1 [reference]		1 [reference]	
Stage 1	11,262 (0.24)	1,336 (0.12)	1.75 (1.62, 1.88)	< .001	1.58 (1.46–1.72)	< 0.001
Stage 2	8,991 (0.19)	1,826 (0.2)	3.31 (3.08, 3.55)	< .001	2.94 (2.7–3.19)	< 0.001
Stage 3	2,119 (0.05)	775 (0.37)	7.48 (6.76, 8.28)	< .001	5.24 (4.42–6.2)	< 0.001
AUMCdb						
No AKI	10,235 (0.69)	605 (0.06)	1 [reference]		1 [reference]	
Stage 1	2,456 (0.16)	384 (0.16)	2.95 (2.57, 3.38)	< .001	2.91 (2.53–3.36)	< 0.001
Stage 2	1,589 (0.11)	428 (0.27)	5.87 (5.11, 6.73)	< .001	5.16 (4.42–6.03)	< 0.001
Stage 3	643 (0.04)	312 (0.49)	15 (12.6, 17.9)	< .001	13.59 (10.49–17.62)	< 0.001

^*1*^Model includes age, weight, gender and whether diagnosed on admission.

All covariates in the adjusted model were significant.

### Secondary outcome—external validation:

The AUMCdb contained 1,573,533 eligible UO records across 18,147 ICU admissions and 16,344 patients. After exclusions, 97.7% of eligible admissions (17,736/18,147) and 94.7% of eligible urine measurements (1,489,527/1,573,533) were included, representing 89,960 days of UO monitoring in the ICU. 14,923 first ICU admissions met the minimal requirements for calculating KDIGO-UO (Supplemental Fig. 3). The durations of collection were shorter (87 [SD, 104] vs. 99 [SD, 176] minutes, p < 0.001), and the measured volumes were lower accordingly (131 [SD, 114] vs. 135 [SD, 144] ml, p < 0.001).

The AUMCdb population differed from MIMICdb in most demographic variables (Table 1). It had a lower proportion of women (33 vs. 44%, p < 0.001), younger average age (63 years [SD, 15] vs. 65 years [SD, 17], p < 0.001), lower sCr at ICU admission (1.21 mg/dL [SD, 1.06] vs 1.35md/dL [SD, 1.37, p < 0.001) and shorter ICU stays (1.3 [IQR, 0.9–4.7] vs. 2.0 [IQR, 1.2–3.8] days, p < 0.001).

As shown in Table 3, total AKI diagnosis rate in the first days and prevalence at admission was lower at AUMCdb (31.4 vs. 48.2%, p < 0.001, and 6.9 vs. 13.8%, p < 0.001, respectively). The peak KDIGO-UO staging in the first days was comparable (52.4 vs. 50.3% [stage 1], 33.9 vs. 40.2% [stage 2], and 13.7 vs. 9.5% [stage 3] for AUMCdb vs. MIMICdb respectively, pooled p < 0.001). In the stratified comparison of ICU admission by maximal KDIGO-UO staging, even though AUMCdb population had more males and lower sCr at admission, admissions with AKI on the first days had worse outcomes in terms of AKI staging, days in ICU, RRT rates and mortality rates (all with p < 0.001) (Table 4). Despite differences, a similar consistent correlation was shown between increased staging and all measured clinical outcomes (all with p < 0.001), 30-day survival (Log-Rank p < 0.001 for each between-group comparison) (Fig. 5) and adjusted and unadjusted 30-day mortality (Table 5).

It is important to note that although adjusted ORs in AUMCdb increase with higher KDIGO-UO staging, this reflects the multiplication of a lower baseline mortality in stage 0 and a smaller proportion of patients reaching these stages. This pattern may indicate a subset of critically ill patients, whereas a larger proportion of less severe cases might progress to these stages in a different setting.

## Discussion

This study presents a reproducible method for handling UO data. We demonstrate UO rate standardization in hourly resolution for more than 85,000 ICU admissions, report oliguric-AKI rates (by UO^cons^ criteria), and conduct 30-day survival and mortality analysis with maximal stage stratification. The identified AKIs and their maximum staging were also consistently associated with acute peak sCr and discharge sCr, RRT rates, ICU and hospital LOS and hospital mortality.

The method was applied to a second database (i.e., external validation). Despite the AUMCdb population having favorable prognostic factors compared to MIMICdb—including younger age, lower mean sCr at ICU admission (despite a higher proportion of males), and shorter ICU stays—RRT use and mortality rates remained similar. While AKI incidence was lower and admission sCr levels were better in AUMCdb, clinical outcomes were worse, with more severe AKI episodes, longer ICU stays, and higher RRT and mortality rates. The association with clinical outcomes remained consistent despite the different baseline characteristics and AKI rates.

Descriptive statistics revealed patterns consistent with valid observations in Fig. 3; the bimodal distribution may be explained by one group of patients with a Gaussian distribution of normal renal physiology and a second group with pathological renal physiology (AKI). R/L nephrostomy production rates were similar and were approximately half of Foley catheter rates. The distributions of the durations of collection for voiding and condom catheter were suggestive of the physiological time to reach spontaneous voiding. The distribution of duration of collections described for straight catheters suggested alleviation of retention. Longer durations of collection were associated with lower rates, suggesting prolonged collection periods in instances of low urine production. These findings were also replicated with the AUMCdb. A comparison of the proposed methodology with simple hourly volume summation showed a difference of at least 50 ml in 33.4% of the computed hours in the derivation cohort and 39.9% in the validation cohort (Supplemental Table 4).

Our suggested use of the UO^cons^ interpretation is more complex; without digital automation, clinicians will be required to perform calculations and comparisons for each hour rather than simply summing the data and averaging it. However, its staging offers better discrimination for all clinical outcomes and a mortality model with a better fit (Supplemental Appendix 3). Additionally, when comparing the UO^cons^ with the UO^mean^, total AKI diagnosis at the first days of admission was reduced by 15–17% (in MIMICdb and AUMCdb, respectively), while mortality rates in the undiagnosed group were increased only by 1–2%, thus achieving increased specificity with minimal impact on sensitivity. However, as demonstrated, the presented method can be used to calculate and compare both interpretations in future research. Therefore, we extend the work of Monard, C et al., who observed similar trends when comparing different KDIGO-UO interpretations and advocated for consensus guidelines^17^.

Using the proposed method can, for example, enable the utilization of meta-analyses across multiple studies and the uniform input of data for training ML models. Along with the suggested standardization and code, it could facilitate subsequent inference using available EHR data to enhance AKI e-alerts, even in its complex interpretation such as UO^cons^. The standardization of hourly UO will contribute to the ability to perform high-resolution retrospective analyses, such as treatments for alleviating fluid overload and oliguria. Finally, continued research on high-resolution UO may shed light on new physiological mechanisms, such as studying pathophysiological differences between AKI that begins with oliguria versus an event that starts with increased sCr, thereby updating classification criteria, identifying more targeted treatments and improving prognosis.

This study is retrospective and, therefore, limited to existing data. Data on collection durations are lacking, and UO documentation is limited to the ICU stay. The lack of prior UO documentation obscures AKI onset and severity at ICU admission, affecting research and clinical practice; Adjusted models should include a “diagnosis at admission” covariate, and clinicians should maintain high suspicion for those patients. The EHR contains ICD codes pertinent only to the time of discharge, restricting cross-referencing against identified AKI events. The ability to retrieve an incidence within another dataset does not necessarily imply that this incidence is true. Finally, isolated episodes of oliguria (not followed by a sCr rise) probably differ from an episode of oliguria followed by a sCr rise. It remains to be elucidated whether the former equates to the latter in terms of prognostic implications and the need to trigger a clinical intervention.

In conclusion, based on simple charting, the method described offers a potentially applicable tool for oliguric-AKI research. It may serve as a platform for future high-resolution UO research, training and inferring using ML models, and improved e-alerts. The method is compatible with publicly available databases and standard EHRs, as well as custom-made Excel tables. The generalized computational code of the method is open-source and available for implementation in further AKI research.

## Supplementary Information

Supplementary Information.

## Data Availability

The datasets analyzed during the current study are available in the MIMIC-IV and Amsterdam-UMC repositories, https://github.com/MIT-LCP/mimic-code^40–44^ and https://github.com/AmsterdamUMC/AmsterdamUMCdb^45^, respectively. The code and comprehensive instructions for recreating the data used in this study, particularly the hourly-adjusted UO and AKI tables, are publicly available on GitHub, https://github.com/arielhasidim/mimic-uo-and-aki and https://github.com/arielhasidim/aumc-uo-and-aki.
