# Supplementary material for: Toward the standardization of big datasets of urine output for AKI analysis: a multicenter validation study
Source: Sci Rep. 2025 Jun 6;15:20009. doi: 10.1038/s41598-025-95535-4 (PMC12144275; doi:10.1038/s41598-025-95535-4)
Supplement: Supplementary file 1 — Supplementary Information. [file 41598_2025_95535_MOESM1_ESM.docx]

Electronic supplementary material

Toward the standardization of big datasets of urine output for AKI analysis: A multicenter validation study

**Supplemental Methods 1.** Steps for the Standardization of Big Datasets

**Supplemental Appendix 1.** Temporal Trends

**Supplemental Appendix 2.** Charting Duplications

**Supplemental Table 1.** Sensitivity Analysis for Excluding Durations of Collection Outliers

**Supplemental Appendix 4.** Age and Weight

**Supplemental Appendix 3.** Comparison of KDIGO-UO Interpretations

**Supplemental Methods 2.** Sample Size and Power Analysis

**Supplemental Figure 1.** Frequency of Urine Output Charting

**Supplemental Table 2.** Durations of Collection for All Volume Measurements

**Supplemental Figure 2.** The Proportion of 'Zero Value' UO Measurements

**Supplemental Table 3.** Durations of Collection for Zero-Volume Measurements

**Supplemental Appendix 5.** Rates vs Durations of Collection

**Supplemental Figure 3.** Flowchart of Urine Data Selection and Adjustment Process in AUMCdb

**Supplemental Table 4.** Comparison of Hourly-Adjustment vs Hourly Summation

# Supplementary Methods 1. Steps for the Standardization of Big Datasets

## Raw Urine Output Data

Raw UO data was obtained from the `OUTPUTEVENTS` table in the MIMIC database and was used to create the raw records table. The official query from the MIMIC-IV repository was used to list all documented UO measurements (1).

The table below includes a real sample of a single patient’s raw UO data that will be used to demonstrate the calculation of hourly-adjusted UO in the next section. This example presents a chronological sequence of rows that includes the time of UO measurement (`CHARTIME`), the measured UO volume in milliliters (`VALUE`), and the source of UO measurement (`ITEMID`). For convenience, we dropped the ICU identifier (`STAY_ID`) and added the label for the source of measurement (`LABEL` for `ITEMID`).

| **Example of Raw Urine Output Charting for a Single Patient** | | | |
| --- | --- | --- | --- |
| CHARTTIME | VALUE | ITEMID | LABEL |
| 2144-05-19 17:00:00 | 50 | 226559 | Foley |
| 2144-05-19 18:00:00 | 20 | 226559 | Foley |
| 2144-05-19 19:00:00 | 20 | 226559 | Foley |
| 2144-05-19 19:46:00 | 150 | 226564 | R Nephrostomy |
| 2144-05-19 20:00:00 | 20 | 226559 | Foley |
| 2144-05-19 21:00:00 | 35 | 226559 | Foley |
| 2144-05-19 22:00:00 | 35 | 226559 | Foley |
| 2144-05-19 22:00:00 | 45 | 226564 | R Nephrostomy |
| 2144-05-20 00:00:00 | 23 | 226559 | Foley |
| 2144-05-20 01:00:00 | 40 | 226559 | Foley |
| 2144-05-20 01:00:00 | 35 | 226564 | R Nephrostomy |
| 2144-05-20 02:00:00 | 17 | 226559 | Foley |
| 2144-05-20 03:00:00 | 22 | 226559 | Foley |
| 2144-05-20 04:00:00 | 20 | 226559 | Foley |
| 2144-05-20 05:00:00 | 50 | 226559 | Foley |

Each row represents a continuous physiological process (i.e., urine production). However, the `CHARTTIME` column contains only discrete timestamps and does not provide information about the duration of urine production (i.e., the collection period). Therefore, our main objective for the raw data analysis was to identify potential durations of collection that would allow for the calculation of the UO rate by dividing the volume by the collection period.

According to the MIMIC documentation, *“It is usually reasonable to assume that any output recorded is for the interval between the current CHARTTIME and the previous CHARTTIME for the same item”* (2). Therefore, the time interval between two consecutive UO measurements may potentially be used as the collection period for the most recent measurement. To ensure accurate interpretation of subsequent records as sequential “back-to-back” measurements, it was necessary to verify that each record in the ICU stay had a distinct `CHARTTIME`, which required addressing potential issues of duplicate charting and variations in urine collection methods beforehand (eAppendix 1 in the Supplement).

The investigation into duplication cases revealed the existence of four potentially different physiologic compartments of urine accumulation: the urinary bladder, right and left nephrostomies, and illeoconduit. Consequently, to evaluate the UO rates for each compartment, the durations of collection were calculated from the last instance recorded for that specific compartment. This approach allowed for the consideration of multiple simultaneous UO rates for a given patient, which could later be aggregated to represent the total hourly-adjusted UO.

The durations of collection between consecutive UO measurements were determined by the time interval between their `CHARTTIMES`. Therefore, the volume measured in the first record for each compartment in each ICU stay was excluded; however, its `CHARTTIME` was retained to calculate the collection period of the subsequent UO measurement.

1. Johnson AEW, Gow B. Source code for: MIMIC Code Repository - Urine Output [Internet]. GitHub. 2023 [cited 2023 Apr 25]. Available from: <https://github.com/MIT-LCP/mimic-code/blob/main/mimic-iv/concepts/measurement/urine_output.sql>
2. MIT-LCP. MIMIC documentation - Inputs and outputs [Internet]. MIMIC. 2015 [cited 2023 Apr 25]. Available from: <https://mimic.mit.edu/docs/iii/about/io/>

## Adjusting for Hourly Urine Output

After calculating and analyzing the durations of collection, hourly UO was adjusted in two steps. In the first step, the UO rate for each collection period was calculated by dividing each UO measurement by its corresponding collection period.

In the second step, the UO was adjusted for each hour of the patient’s ICU stay. The first and last UO measurements in ICU stay were identified, and the start and end times were determined by rounding down the `CHARTTIME` of the first UO measurement and rounding up the `CHARTTIME` of the last UO measurement. These times were then used to create a list of the hourly intervals for each ICU stay. Next, the UO rates that overlapped with each hourly interval were identified, and the proportion of overlap within that hour was noted. Finally, the hourly-adjusted UO rate was calculated by summing the volumes of the overlapping portions of UO rates for each hour. An adjusted value was only calculated when a UO rate was present for most of the hour.

The figure below shows the entire process of calculating the hourly-adjusted UO for the patient from the example in the table from the previous section. For comparison with the hourly-adjusted UO, we included a simple hourly sum of urine volume records at the bottom of the figure.

### From Raw to Hourly-Adjusted Urine Output

# Supplementary Appendix 1. Temporal Trends

The MIMIC-IV dataset encompasses information collected between 2008 and 2019 (1). Each patient in the dataset has an “anchor year group” organized into three-year periods. For each hospital admission, we adjusted the beginning of the anchor year group based on the years elapsed since the patient’s first hospital admission.


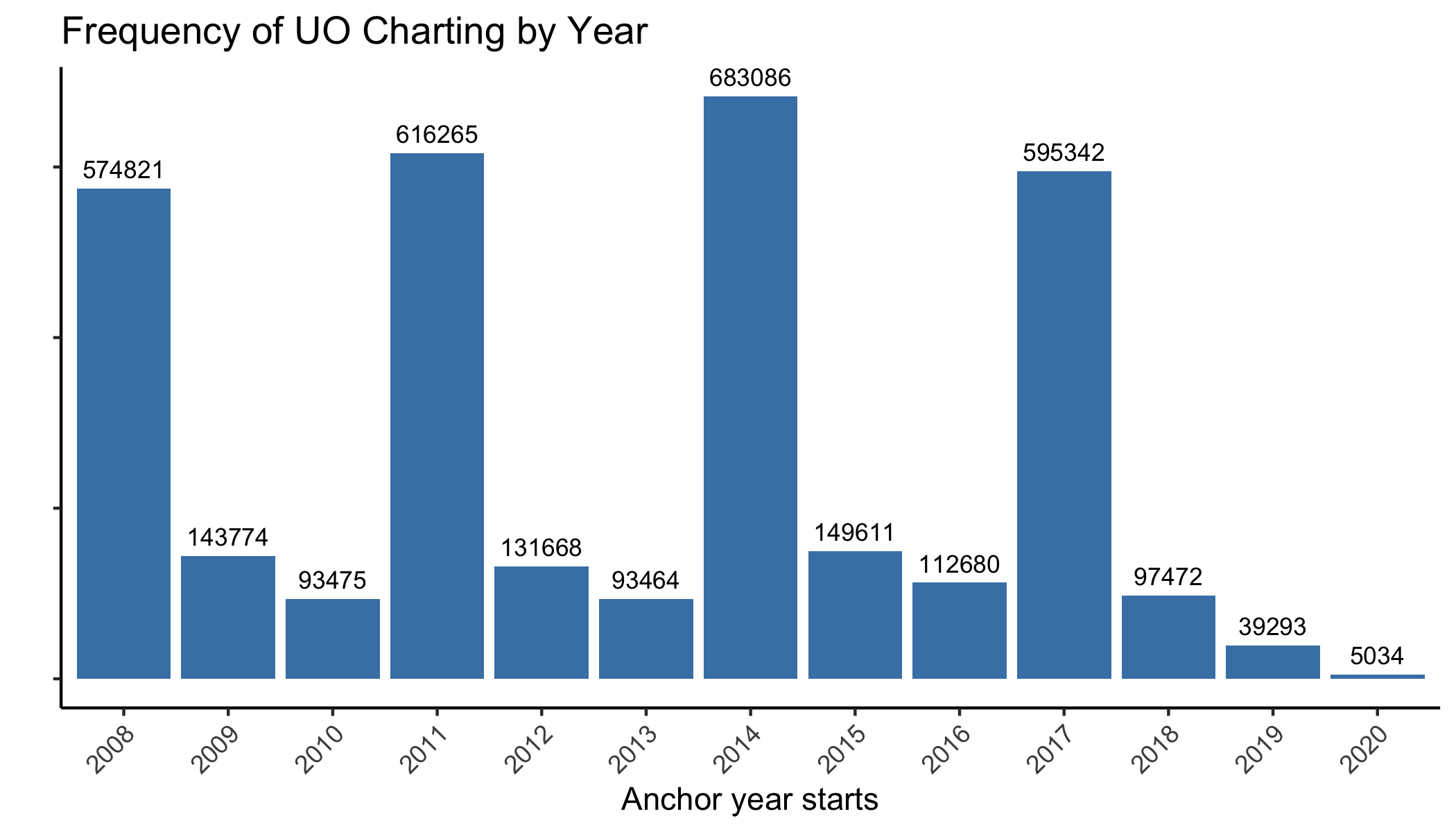


To facilitate the analysis, the data was grouped into 3-year intervals based on the observed distribution of records. Subsequent analyses will follow this grouping structure.


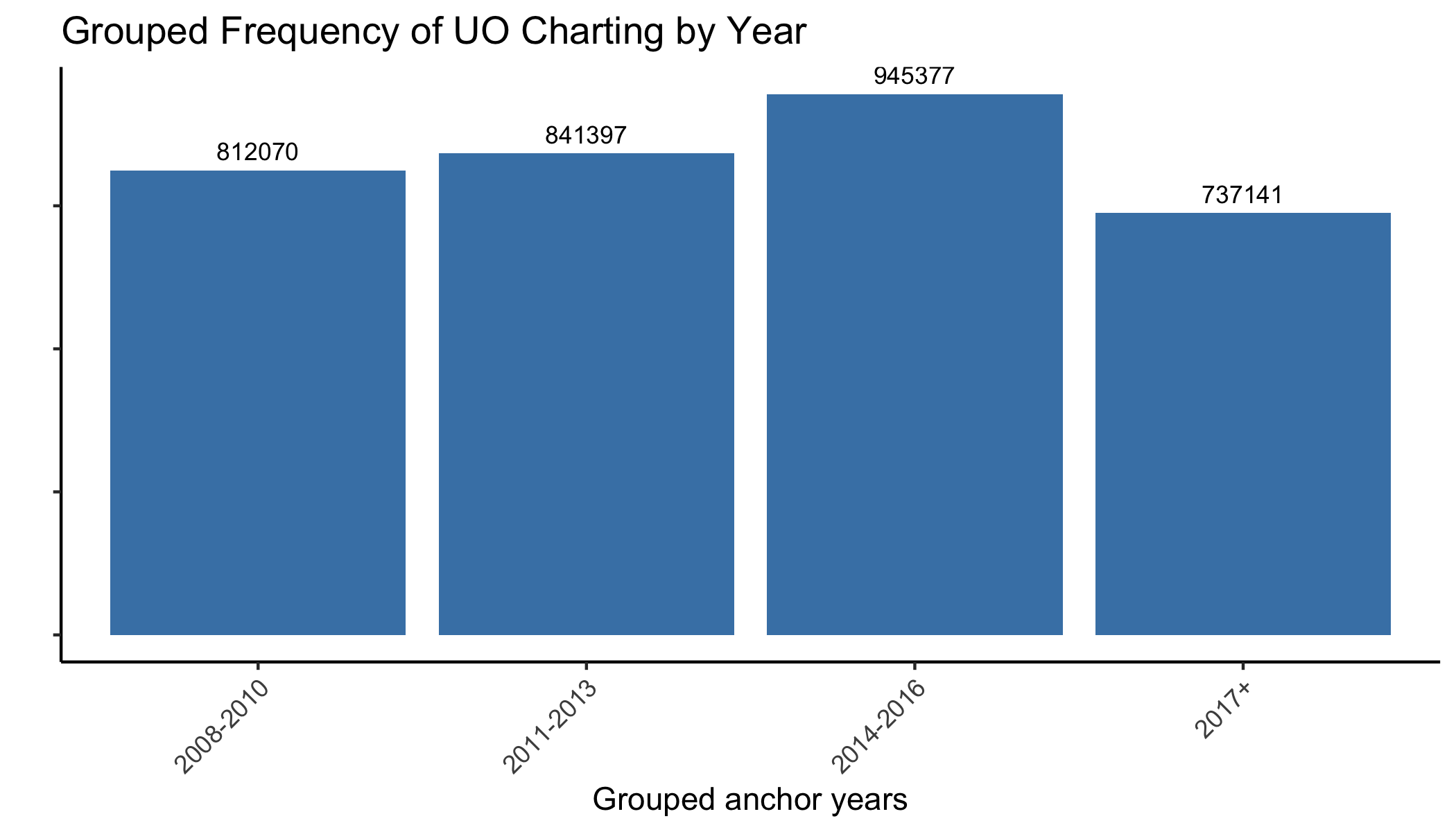


As shown in the figure below, the distribution of sources used for urine output monitoring has changed over the years. Notably, the rates of spontaneous voiding, condom catheterization, and straight catheterization have all increased.


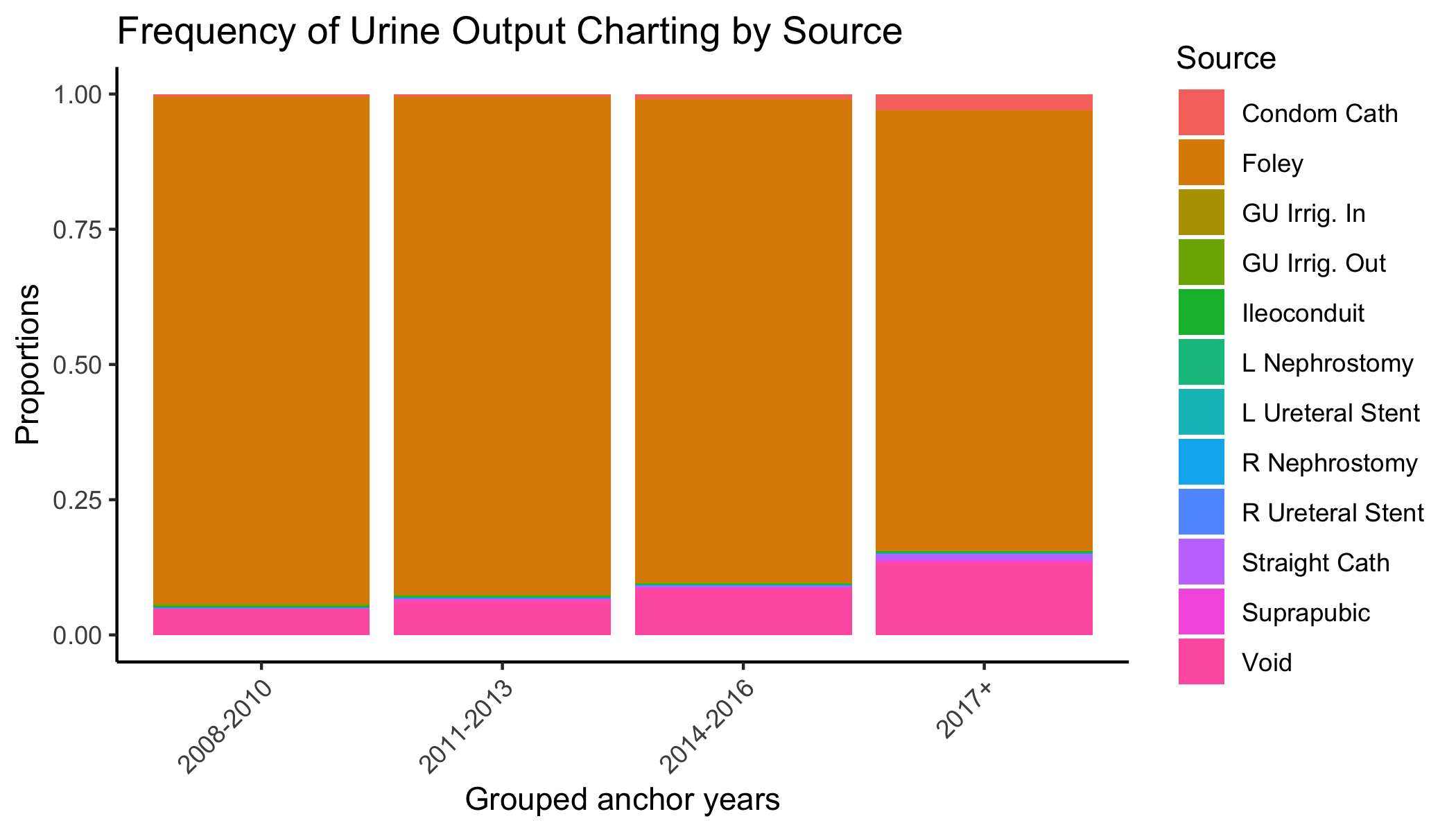


GU Irrig: Genitourinary Irrigation; L: Left; R: Right.

The next figure shows that the sources of UO charting for Foley catheter and spontaneous voiding, which account for over 97.3% of all records, have remained consistent over time. However, a clear trend can be seen for straight catheter, which includes shorter durations of collection and higher average measured volumes. The variability observed in the remaining sources does not show a clear trend and is consistent with the expected natural variability seen in datasets with relatively fewer measurements. This occurs because each of the sources accounts for only a fraction of a percentage of the total measurements.


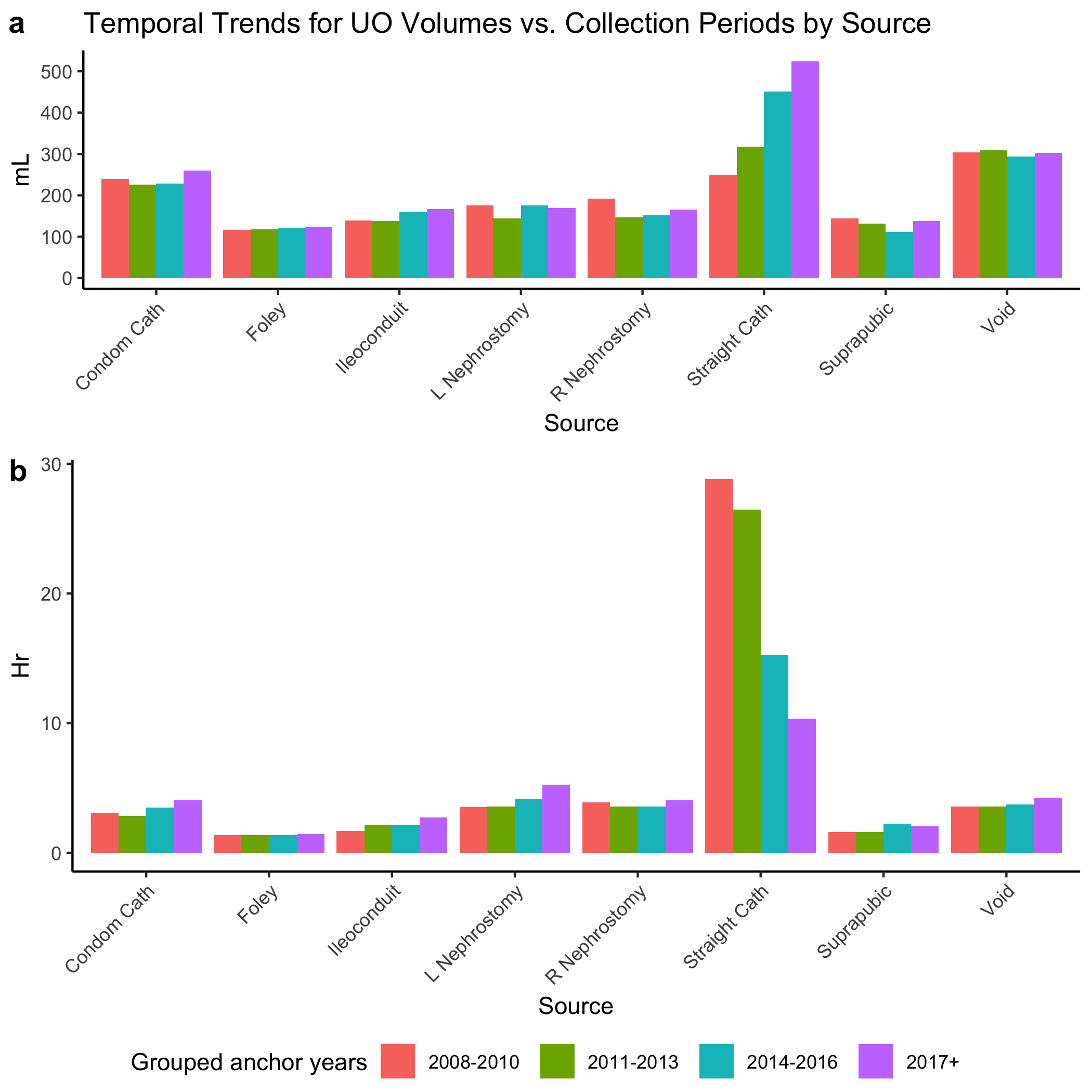


Urine output charting and collection time calculations after exclusion. (a) Mean urine output volume; (b) Mean collection time. L: Left; R: Right.

1. Johnson AEW, Bulgarelli L, Shen L, Gayles A, Shammout A, Horng S, et al. MIMIC-IV, a freely accessible electronic health record dataset. Sci Data. 2023;10:1.

# Supplementary Appendix 2. Charting Duplications

First, we verified that all UO chartings for each patient are distinct by their item and chart time (all rows: 3335985 = distinct rows: 3335985); This ensured the absence of duplicate records from a single source for a patient (without accounting for its volume). Subsequently, a duplication analysis was conducted. To do this, we compiled all the cases of UO measurements documented simultaneously for the same patient from different sources. We then checked for potential errors in record-keeping by verifying whether the values were equal. We found that most records had different values and concluded that duplicate record-keeping errors were unlikely. There were 12,569 documented duplicates, of which 518 had the same volume.

The most common duplicate entries were GU irrigation IN and OUT, which had 5,347 records, accounting for 44.5% of the 12,569 duplicates. These GU irrigants were excluded from our research. We also observed many duplicate entries in combination with nephrostomy, ileoconduit, and urethral catheter. Based on this analysis, we concluded that there are four potential physiologic compartments for the accumulation and drainage of urine output that can be measured. This means that when assuming output events occurred consecutively (“back-to-back”) and using the time interval from the last charted time to define the collection period, it is essential to assess each compartment individually.

| **Simultaneous UO Charting and Corresponding Source Count by Volume Equality** | | |
| --- | --- | --- |
| **Characteristic** | **Different volume** N = 12,051*^1^* | **Equal volume** N = 518*^1^* |
| GU Irrigant Volume In,GU Irrigant/Urine Volume Out | 4,189 (35%) | 8 (1.5%) |
| Foley,L Nephrostomy | 1,127 (9.4%) | 69 (13%) |
| Foley,GU Irrigant Volume In,GU Irrigant/Urine Volume Out | 1,158 (9.6%) | 3 (0.6%) |
| Foley,R Nephrostomy | 1,077 (8.9%) | 60 (12%) |
| R Nephrostomy,L Nephrostomy | 988 (8.2%) | 125 (24%) |
| Foley,Void | 703 (5.8%) | 61 (12%) |
| Foley,Suprapubic | 557 (4.6%) | 55 (11%) |
| R Nephrostomy,Ileoconduit | 346 (2.9%) | 24 (4.6%) |
| Foley,R Nephrostomy,L Nephrostomy | 322 (2.7%) | 4 (0.8%) |
| Foley,GU Irrigant Volume In | 186 (1.5%) | 1 (0.2%) |
| Void,Straight Cath | 161 (1.3%) | 4 (0.8%) |
| Suprapubic,R Nephrostomy | 151 (1.3%) | 11 (2.1%) |
| Foley,Condom Cath | 113 (0.9%) | 18 (3.5%) |
| R Ureteral Stent,Foley | 122 (1.0%) | 8 (1.5%) |
| Suprapubic,L Nephrostomy | 120 (1.0%) | 9 (1.7%) |
| Void,Condom Cath | 78 (0.6%) | 14 (2.7%) |
| L Nephrostomy,Ileoconduit | 90 (0.7%) | 1 (0.2%) |
| R Ureteral Stent,L Ureteral Stent | 51 (0.4%) | 7 (1.4%) |
| Void,L Nephrostomy | 48 (0.4%) | 7 (1.4%) |
| Condom Cath,Straight Cath | 47 (0.4%) | 2 (0.4%) |
| Foley,Ileoconduit | 46 (0.4%) | 2 (0.4%) |
| R Ureteral Stent,L Nephrostomy | 45 (0.4%) | 2 (0.4%) |
| Condom Cath,R Nephrostomy | 29 (0.2%) | 3 (0.6%) |
| R Nephrostomy,L Nephrostomy,Ileoconduit | 29 (0.2%) | 0 (0%) |
| Void,R Nephrostomy | 23 (0.2%) | 3 (0.6%) |
| Condom Cath,Suprapubic | 21 (0.2%) | 2 (0.4%) |
| Foley,Straight Cath | 17 (0.1%) | 4 (0.8%) |
| Foley,GU Irrigant/Urine Volume Out | 19 (0.2%) | 0 (0%) |
| R Ureteral Stent,L Ureteral Stent,L Nephrostomy | 19 (0.2%) | 0 (0%) |
| R Ureteral Stent,R Nephrostomy,Ileoconduit | 16 (0.1%) | 0 (0%) |
| L Ureteral Stent,Foley | 14 (0.1%) | 1 (0.2%) |
| R Ureteral Stent,L Ureteral Stent,Foley | 14 (0.1%) | 0 (0%) |
| R Ureteral Stent,R Nephrostomy | 9 (<0.1%) | 4 (0.8%) |
| Suprapubic,R Nephrostomy,L Nephrostomy | 12 (<0.1%) | 1 (0.2%) |
| Condom Cath,R Nephrostomy,L Nephrostomy | 11 (<0.1%) | 0 (0%) |
| Condom Cath,L Nephrostomy | 8 (<0.1%) | 2 (0.4%) |
| Foley,Suprapubic,R Nephrostomy | 10 (<0.1%) | 0 (0%) |
| Foley,L Nephrostomy,GU Irrigant Volume In | 7 (<0.1%) | 0 (0%) |
| Foley,L Nephrostomy,GU Irrigant Volume In,GU Irrigant/Urine Volume Out | 6 (<0.1%) | 0 (0%) |
| R Nephrostomy,L Nephrostomy,GU Irrigant Volume In,GU Irrigant/Urine Volume Out | 6 (<0.1%) | 0 (0%) |
| L Nephrostomy,GU Irrigant Volume In,GU Irrigant/Urine Volume Out | 5 (<0.1%) | 0 (0%) |
| L Ureteral Stent,R Nephrostomy,L Nephrostomy | 5 (<0.1%) | 0 (0%) |
| Void,R Nephrostomy,L Nephrostomy | 5 (<0.1%) | 0 (0%) |
| R Ureteral Stent,Ileoconduit | 4 (<0.1%) | 0 (0%) |
| Void,Suprapubic | 4 (<0.1%) | 0 (0%) |
| Foley,L Nephrostomy,Ileoconduit | 3 (<0.1%) | 0 (0%) |
| Foley,Void,Condom Cath | 1 (<0.1%) | 1 (0.2%) |
| Ileoconduit,GU Irrigant Volume In,GU Irrigant/Urine Volume Out | 2 (<0.1%) | 0 (0%) |
| R Nephrostomy,L Nephrostomy,GU Irrigant Volume In | 2 (<0.1%) | 0 (0%) |
| R Ureteral Stent,Foley,R Nephrostomy | 2 (<0.1%) | 0 (0%) |
| R Ureteral Stent,Void | 2 (<0.1%) | 0 (0%) |
| Suprapubic,GU Irrigant Volume In,GU Irrigant/Urine Volume Out | 2 (<0.1%) | 0 (0%) |
| Suprapubic,Ileoconduit | 1 (<0.1%) | 1 (0.2%) |
| Void,Ileoconduit | 2 (<0.1%) | 0 (0%) |
| Foley,R Nephrostomy,GU Irrigant Volume In | 1 (<0.1%) | 0 (0%) |
| Foley,R Nephrostomy,Ileoconduit | 1 (<0.1%) | 0 (0%) |
| L Nephrostomy,Straight Cath | 1 (<0.1%) | 0 (0%) |
| L Ureteral Stent,Foley,L Nephrostomy | 1 (<0.1%) | 0 (0%) |
| L Ureteral Stent,L Nephrostomy | 1 (<0.1%) | 0 (0%) |
| L Ureteral Stent,Suprapubic | 1 (<0.1%) | 0 (0%) |
| R Nephrostomy,GU Irrigant Volume In,GU Irrigant/Urine Volume Out | 1 (<0.1%) | 0 (0%) |
| R Nephrostomy,GU Irrigant/Urine Volume Out | 1 (<0.1%) | 0 (0%) |
| R Nephrostomy,L Nephrostomy,GU Irrigant/Urine Volume Out | 1 (<0.1%) | 0 (0%) |
| R Nephrostomy,L Nephrostomy,Straight Cath | 1 (<0.1%) | 0 (0%) |
| R Ureteral Stent,L Ureteral Stent,Foley,Suprapubic | 1 (<0.1%) | 0 (0%) |
| R Ureteral Stent,Straight Cath | 1 (<0.1%) | 0 (0%) |
| Suprapubic,GU Irrigant Volume In | 1 (<0.1%) | 0 (0%) |
| Suprapubic,GU Irrigant/Urine Volume Out | 0 (0%) | 1 (0.2%) |
| Void,Condom Cath,Straight Cath | 1 (<0.1%) | 0 (0%) |
| Void,GU Irrigant Volume In | 1 (<0.1%) | 0 (0%) |
| Void,GU Irrigant Volume In,GU Irrigant/Urine Volume Out | 1 (<0.1%) | 0 (0%) |
| *^1^*n (%) | | |

# Supplementary Table 1. Sensitivity Analysis for Excluding Durations of Collection Outliers

|  | **MIMICdb** | | | | | | | **AUMCdb** | | | | | | |
| --- | --- | --- | --- | --- | --- | --- | --- | --- | --- | --- | --- | --- | --- | --- |
| **Characteristic** | **N** | **95th prec. for rate bellow 20th prec.** N = 9,803*^1^* | **N** | **99th prec. for rate bellow 20th prec.** N = 10,658*^1^* | **N** | **No exclusion** N = 11,111*^1^* | **p-value***^2^* | **N** | **95th prec. for rate bellow 20th prec.** N = 2,107*^1^* | **N** | **99th prec. for rate bellow 20th prec.** N = 2,204*^1^* | **N** | **No exclusion** N = 2,233*^1^* | **p-value***^2^* |
| Age at Hospital Admission, years | 9,803 | 67 (16) | 10,658 | 67 (16) | 11,111 | 67 (16) | 0.9 | 2,107 | 67 (14) | 2,204 | 67 (14) | 2,233 | 67 (14) | >0.9 |
| Weight at ICU Admission, kg | 9,803 | 89 (27) | 10,658 | 89 (27) | 11,111 | 88 (27) | 0.036 | 2,107 | 85 (15) | 2,204 | 84 (15) | 2,233 | 84 (15) | >0.9 |
| Gender | 9,803 |  | 10,658 |  | 11,111 |  | >0.9 | 2,080 |  | 2,175 |  | 2,204 |  | >0.9 |
| F |  | 4,362 (44%) |  | 4,745 (45%) |  | 4,950 (45%) |  |  | 733 (35%) |  | 770 (35%) |  | 776 (35%) |  |
| M |  | 5,441 (56%) |  | 5,913 (55%) |  | 6,161 (55%) |  |  | 1,347 (65%) |  | 1,405 (65%) |  | 1,428 (65%) |  |
| Ethnicity | 8,427 |  | 9,142 |  | 9,527 |  | >0.9 |  |  |  |  |  |  |  |
| African American |  | 967 (11%) |  | 1,048 (11%) |  | 1,084 (11%) |  |  |  |  |  |  |  |  |
| Asian |  | 169 (2.0%) |  | 193 (2.1%) |  | 205 (2.2%) |  |  |  |  |  |  |  |  |
| Caucasian |  | 6,632 (79%) |  | 7,186 (79%) |  | 7,497 (79%) |  |  |  |  |  |  |  |  |
| Hispanic |  | 279 (3.3%) |  | 299 (3.3%) |  | 310 (3.3%) |  |  |  |  |  |  |  |  |
| Other |  | 380 (4.5%) |  | 416 (4.6%) |  | 431 (4.5%) |  |  |  |  |  |  |  |  |
| CCI Score | 9,803 | 5 (3, 7) | 10,658 | 5 (3, 7) | 11,111 | 5 (3, 7) | 0.8 |  |  |  |  |  |  |  |
| CKD, Stage 1-4 | 9,799 | 2,332 (24%) | 10,654 | 2,582 (24%) | 11,107 | 2,678 (24%) | 0.8 |  |  |  |  |  |  |  |
| Diabetes Mellitus | 9,799 | 2,438 (25%) | 10,654 | 2,622 (25%) | 11,107 | 2,710 (24%) | 0.7 |  |  |  |  |  |  |  |
| SOFA Score at ICU Admission | 9,803 | 5 (2, 8) | 10,658 | 5 (2, 8) | 11,111 | 5 (2, 8) | 0.6 |  |  |  |  |  |  |  |
| SAPS-II at ICU Admission | 9,743 | 39 (30, 50) | 10,590 | 39 (30, 50) | 11,041 | 39 (29, 50) | 0.6 |  |  |  |  |  |  |  |
| APS-III Score at ICU Admission | 9,803 | 46 (34, 63) | 10,658 | 46 (34, 64) | 11,111 | 46 (34, 63) | 0.6 |  |  |  |  |  |  |  |
| First Creatinine in ICU, mg/dL | 9,775 | 1.66 (1.83) | 10,621 | 1.70 (1.91) | 11,074 | 1.70 (1.91) | 0.8 | 2,105 | 1.67 (1.79) | 2,202 | 1.71 (1.85) | 2,231 | 1.72 (1.87) | 0.8 |
| Peak Creatinine at first days, mg/dL | 9,771 | 2.07 (2.12) | 10,617 | 2.11 (2.19) | 11,065 | 2.10 (2.19) | 0.6 | 2,095 | 2.43 (1.86) | 2,191 | 2.45 (1.90) | 2,219 | 2.45 (1.91) | >0.9 |
| ICU Discharge Creatinine, mg/dL | 9,775 | 1.68 (1.71) | 10,621 | 1.70 (1.75) | 11,074 | 1.69 (1.74) | 0.7 | 2,105 | 1.66 (1.34) | 2,202 | 1.67 (1.36) | 2,231 | 1.67 (1.35) | >0.9 |
| Peak KDIGO-Cr at first days | 9,737 |  | 10,577 |  | 11,015 |  | >0.9 |  |  |  |  |  |  |  |
| 0 |  | 5,578 (57%) |  | 6,039 (57%) |  | 6,357 (58%) |  |  |  |  |  |  |  |  |
| 1 |  | 2,557 (26%) |  | 2,805 (27%) |  | 2,887 (26%) |  |  |  |  |  |  |  |  |
| 2 |  | 715 (7.3%) |  | 759 (7.2%) |  | 773 (7.0%) |  |  |  |  |  |  |  |  |
| 3 |  | 887 (9.1%) |  | 974 (9.2%) |  | 998 (9.1%) |  |  |  |  |  |  |  |  |
| Time in hospital, days | 9,803 | 8 (5, 14) | 10,658 | 8 (5, 14) | 11,111 | 8 (5, 14) | 0.5 |  |  |  |  |  |  |  |
| Time in ICU, days | 9,803 | 3.1 (1.9, 5.7) | 10,658 | 3.1 (1.9, 5.7) | 11,111 | 3.1 (1.9, 5.7) | 0.9 | 2,107 | 5 (3, 13) | 2,204 | 5 (2, 13) | 2,233 | 5 (2, 13) | >0.9 |
| Renal replacement therapy | 9,803 | 1,345 (14%) | 10,658 | 1,519 (14%) | 11,111 | 1,574 (14%) | 0.5 | 2,107 | 646 (31%) | 2,204 | 687 (31%) | 2,233 | 696 (31%) | >0.9 |
| Hospital Mortality | 9,803 | 1,805 (18%) | 10,658 | 2,031 (19%) | 11,111 | 2,111 (19%) | 0.4 | 2,107 | 654 (31%) | 2,204 | 689 (31%) | 2,233 | 698 (31%) | >0.9 |
| *^1^*Mean (SD); n (%); Median (Q1, Q3) | | | | | | | | | | | | | | |
| *^2^*Kruskal-Wallis rank sum test; Pearson's Chi-squared test | | | | | | | | | | | | | | |
| Studying the potential effect of the inclusion of exceptional prolonged durations of collection by comparing the impact of different thresholds for excluding outliers on identification of ICU stays with oliguric-AKI stage ≥ 2 on the first 72 hours of admission. | | | | | | | | | | | | | | |

# Supplementary Appendix 3. Comparison of KDIGO-UO Interpretations

This is a comparison between different interpretations of KDIGO-UO criteria:

- **UOmean** - where the average ml/kg/hr over 6, 12, and 24-hour windows meet the threshold.
- **UOcons** - where UO meets KDIGO’s threshold in each consecutive hour.
- **Block Summation** - where “blind” summation over the relevant hourly windows meets the threshold (MIMIC repo. official derivation), *only for MIMICdb*

The next table demonstrates the variability in rates of diagnosis and maximal staging of oliguric-AKI according to the different interpretations of KDIGO-UO criteria; All were found to be statistically significant. UOcons is generally more conservative in both diagnosis and staging.

|  | **MIMICdb** | | | | **AUMCdb** | | |
| --- | --- | --- | --- | --- | --- | --- | --- |
| **Characteristic** | **Block summation** N = 46,115*^1^* | **UO-Average** N = 46,344*^1^* | **UO-Consecutive** N = 46,344*^1^* | **p-value***^2^* | **UO-Average** N = 14,923*^1^* | **UO-Consecutive** N = 14,923*^1^* | **p-value***^2^* |
| Oliguric-AKI on the first days | 27,188 (59.0%) | 29,385 (63.4%) | 22,372 (48.3%) | <0.001 | 7,429 (49.8%) | 4,688 (31.4%) | <0.001 |
| Maximum KDIGO staging |  |  |  | <0.001 |  |  | <0.001 |
| 1 | 8,248 (30.3%) | 9,204 (31.3%) | 11,262 (50.3%) |  | 2,509 (33.8%) | 2,456 (52.4%) |  |
| 2 | 14,830 (54.5%) | 15,255 (51.9%) | 8,991 (40.2%) |  | 3,741 (50.4%) | 1,589 (33.9%) |  |
| 3 | 4,110 (15.1%) | 4,926 (16.8%) | 2,119 (9.47%) |  | 1,179 (15.9%) | 643 (13.7%) |  |
| Prevalence at admission | 7,321 (15.9%) | 10,511 (22.7%) | 6,388 (13.8%) | <0.001 | 1,962 (13.1%) | 1,024 (6.86%) | <0.001 |
| *^1^*n (%) | | | | | | | |
| *^2^*Pearson's Chi-squared test | | | | | | | |
| *In post-hoc testing, p<0.001 has been demosntrated for each couple in MIMICdb | | | | | | | |
| Maximal staging was calculated for the first 72 hours of admission. | | | | | | | |

The next table also demonstrates the same variability, now in the diagnosed demographics and clinical outcomes. UOcons diagnosed patients are generally more severe and have worse outcomes.

| **Characteristics of ICU Stays With Oliguric-AKI stage ≥ 2 on the First 72 Hours of Admission by Different Interpretations of KDIGO-UO Criteria** | | | | | | | | | | | | | | | | | | | | | | |
| --- | --- | --- | --- | --- | --- | --- | --- | --- | --- | --- | --- | --- | --- | --- | --- | --- | --- | --- | --- | --- | --- | --- |
|  | **MIMICdb** | | | | | | | | | | | | | | | **AUMCdb** | | | | | | |
| **Characteristic** | **N** | **UO-Average** N = 20,181*^1^* | **N** | **UO-Consecutive** N = 11,110*^1^* | **p-value***^2^* | **N** | **Block summation** N = 18,940*^1^* | **N** | **UO-Consecutive** N = 11,110*^1^* | **p-value***^2^* | **N** | **Block summation** N = 18,940*^1^* | **N** | **UO-Average** N = 20,181*^1^* | **p-value***^2^* | | **N** | **UO-Average** N = 4,920*^1^* | **N** | **UO-Consecutive** N = 2,232*^1^* | **p-value***^2^* |  |
| Age at Hospital Admission, years | 20,181 | 68 (16) | 11,110 | 67 (16) | 0.4 | 18,940 | 68 (16) | 11,110 | 67 (16) | 0.002 | 18,940 | 68 (16) | 20,181 | 68 (16) | 0.006 | | 4,920 | 66 (15) | 2,232 | 67 (14) | 0.002 |  |
| Weight at ICU Admission, kg | 20,181 | 87 (25) | 11,110 | 88 (27) | <0.001 | 18,940 | 88 (25) | 11,110 | 88 (27) | 0.2 | 18,940 | 88 (25) | 20,181 | 87 (25) | 0.011 | | 4,920 | 84 (15) | 2,232 | 84 (15) | 0.057 |  |
| Gender | 20,181 |  | 11,110 |  | 0.028 | 18,940 |  | 11,110 |  | 0.005 | 18,940 |  | 20,181 |  | 0.5 | | 4,845 |  | 2,203 |  | 0.8 |  |
| F |  | 8,731 (43%) |  | 4,950 (45%) |  |  | 8,125 (43%) |  | 4,950 (45%) |  |  | 8,125 (43%) |  | 8,731 (43%) |  | |  | 1,718 (35%) |  | 775 (35%) |  |  |
| M |  | 11,450 (57%) |  | 6,160 (55%) |  |  | 10,815 (57%) |  | 6,160 (55%) |  |  | 10,815 (57%) |  | 11,450 (57%) |  | |  | 3,127 (65%) |  | 1,428 (65%) |  |  |
| Ethnicity | 17,294 |  | 9,526 |  | 0.009 | 16,197 |  | 9,526 |  | <0.001 | 16,197 |  | 17,294 |  | 0.8 | |  |  |  |  |  |  |
| African American |  | 1,734 (10%) |  | 1,084 (11%) |  |  | 1,580 (9.8%) |  | 1,084 (11%) |  |  | 1,580 (9.8%) |  | 1,734 (10%) |  | |  |  |  |  |  |  |
| Asian |  | 373 (2.2%) |  | 205 (2.2%) |  |  | 333 (2.1%) |  | 205 (2.2%) |  |  | 333 (2.1%) |  | 373 (2.2%) |  | |  |  |  |  |  |  |
| Caucasian |  | 13,878 (80%) |  | 7,496 (79%) |  |  | 13,081 (81%) |  | 7,496 (79%) |  |  | 13,081 (81%) |  | 13,878 (80%) |  | |  |  |  |  |  |  |
| Hispanic |  | 524 (3.0%) |  | 310 (3.3%) |  |  | 485 (3.0%) |  | 310 (3.3%) |  |  | 485 (3.0%) |  | 524 (3.0%) |  | |  |  |  |  |  |  |
| Other |  | 785 (4.5%) |  | 431 (4.5%) |  |  | 718 (4.4%) |  | 431 (4.5%) |  |  | 718 (4.4%) |  | 785 (4.5%) |  | |  |  |  |  |  |  |
| CCI Score | 20,181 | 5 (3, 7) | 11,110 | 5 (3, 7) | <0.001 | 18,940 | 5 (3, 7) | 11,110 | 5 (3, 7) | <0.001 | 18,940 | 5 (3, 7) | 20,181 | 5 (3, 7) | 0.11 | |  |  |  |  |  |  |
| CKD, Stage 1-4 | 20,175 | 4,100 (20%) | 11,106 | 2,678 (24%) | <0.001 | 18,934 | 3,910 (21%) | 11,106 | 2,678 (24%) | <0.001 | 18,934 | 3,910 (21%) | 20,175 | 4,100 (20%) | 0.4 | |  |  |  |  |  |  |
| Diabetes Mellitus | 20,175 | 4,950 (25%) | 11,106 | 2,709 (24%) | 0.8 | 18,934 | 4,736 (25%) | 11,106 | 2,709 (24%) | 0.2 | 18,934 | 4,736 (25%) | 20,175 | 4,950 (25%) | 0.3 | |  |  |  |  |  |  |
| SOFA Score at ICU Admission | 20,181 | 4 (2, 7) | 11,110 | 5 (2, 8) | <0.001 | 18,940 | 5 (2, 7) | 11,110 | 5 (2, 8) | 0.021 | 18,940 | 5 (2, 7) | 20,181 | 4 (2, 7) | <0.001 | |  |  |  |  |  |  |
| SAPS-II at ICU Admission | 20,075 | 37 (28, 46) | 11,040 | 39 (29, 50) | <0.001 | 18,865 | 37 (29, 47) | 11,040 | 39 (29, 50) | <0.001 | 18,865 | 37 (29, 47) | 20,075 | 37 (28, 46) | <0.001 | |  |  |  |  |  |  |
| APS-III Score at ICU Admission | 20,181 | 42 (32, 58) | 11,110 | 46 (34, 63) | <0.001 | 18,940 | 43 (32, 59) | 11,110 | 46 (34, 63) | <0.001 | 18,940 | 43 (32, 59) | 20,181 | 42 (32, 58) | <0.001 | |  |  |  |  |  |  |
| First Creatinine in ICU, mg/dL | 20,128 | 1.46 (1.57) | 11,073 | 1.70 (1.92) | <0.001 | 18,897 | 1.46 (1.51) | 11,073 | 1.70 (1.92) | <0.001 | 18,897 | 1.46 (1.51) | 20,128 | 1.46 (1.57) | 0.034 | | 4,914 | 1.40 (1.38) | 2,230 | 1.72 (1.86) | <0.001 |  |
| Peak Creatinine at first days, mg/dL | 20,114 | 1.75 (1.80) | 11,064 | 2.10 (2.19) | <0.001 | 18,889 | 1.76 (1.75) | 11,064 | 2.10 (2.19) | <0.001 | 18,889 | 1.76 (1.75) | 20,114 | 1.75 (1.80) | <0.001 | | 4,882 | 1.79 (1.53) | 2,218 | 2.45 (1.90) | <0.001 |  |
| ICU Discharge Creatinine, mg/dL | 20,128 | 1.42 (1.45) | 11,073 | 1.69 (1.74) | <0.001 | 18,897 | 1.44 (1.44) | 11,073 | 1.69 (1.74) | <0.001 | 18,897 | 1.44 (1.44) | 20,128 | 1.42 (1.45) | 0.009 | | 4,914 | 1.31 (1.08) | 2,230 | 1.67 (1.35) | <0.001 |  |
| Peak KDIGO-Cr at first days | 20,027 |  | 11,014 |  | <0.001 | 18,811 |  | 11,014 |  | <0.001 | 18,811 |  | 20,027 |  | 0.006 | |  |  |  |  |  |  |
| 0 |  | 12,966 (65%) |  | 6,357 (58%) |  |  | 11,860 (63%) |  | 6,357 (58%) |  |  | 11,860 (63%) |  | 12,966 (65%) |  | |  |  |  |  |  |  |
| 1 |  | 4,794 (24%) |  | 2,886 (26%) |  |  | 4,713 (25%) |  | 2,886 (26%) |  |  | 4,713 (25%) |  | 4,794 (24%) |  | |  |  |  |  |  |  |
| 2 |  | 1,126 (5.6%) |  | 773 (7.0%) |  |  | 1,132 (6.0%) |  | 773 (7.0%) |  |  | 1,132 (6.0%) |  | 1,126 (5.6%) |  | |  |  |  |  |  |  |
| 3 |  | 1,141 (5.7%) |  | 998 (9.1%) |  |  | 1,106 (5.9%) |  | 998 (9.1%) |  |  | 1,106 (5.9%) |  | 1,141 (5.7%) |  | |  |  |  |  |  |  |
| Time in hospital, days | 20,181 | 8 (5, 13) | 11,110 | 8 (5, 14) | 0.057 | 18,940 | 8 (5, 14) | 11,110 | 8 (5, 14) | 0.3 | 18,940 | 8 (5, 14) | 20,181 | 8 (5, 13) | <0.001 | |  |  |  |  |  |  |
| Time in ICU, days | 20,181 | 2.9 (1.8, 5.1) | 11,110 | 3.1 (1.9, 5.7) | <0.001 | 18,940 | 2.9 (1.8, 5.2) | 11,110 | 3.1 (1.9, 5.7) | <0.001 | 18,940 | 2.9 (1.8, 5.2) | 20,181 | 2.9 (1.8, 5.1) | 0.13 | | 4,920 | 4 (2, 10) | 2,232 | 5 (2, 13) | <0.001 |  |
| Renal replacement therapy | 20,181 | 1,771 (8.8%) | 11,110 | 1,574 (14%) | <0.001 | 18,940 | 1,655 (8.7%) | 11,110 | 1,574 (14%) | <0.001 | 18,940 | 1,655 (8.7%) | 20,181 | 1,771 (8.8%) | 0.9 | | 4,920 | 745 (15%) | 2,232 | 696 (31%) | <0.001 |  |
| Hospital Mortality | 20,181 | 2,912 (14%) | 11,110 | 2,111 (19%) | <0.001 | 18,940 | 2,859 (15%) | 11,110 | 2,111 (19%) | <0.001 | 18,940 | 2,859 (15%) | 20,181 | 2,912 (14%) | 0.064 | | 4,920 | 963 (20%) | 2,232 | 697 (31%) | <0.001 |  |
| *^1^*Mean (SD); n (%); Median (Q1, Q3) | | | | | | | | | | | | | | | | | | | | | | |
| *^2^*Wilcoxon rank sum test; Pearson's Chi-squared test | | | | | | | | | | | | | | | | | | | | | | |

The next table shows the association of the diagnosis and maximal staging with 30-day mortality OR; It also contains the rates of detection for each stage (including stage 0, ‘no-aki’) and its observed rates of mortality.

| Criteria / Stage |  |  | Unadjusted OR | | Adjusted Model*^1^* | |
| --- | --- | --- | --- | --- | --- | --- |
|  | Patients, No. (%) | Mortality, No. (%) | OR (95% CI) | P value | OR (95% CI) | P value |
| **MIMICdb** | | | | | | |
| UO-Consecutive | | | | | | |
| 0 | 23972 (0.52) | 1715 (0.07) | 1 [Reference] | NA | 1 [Reference] | NA |
| 1 | 11262 (0.24) | 1336 (0.12) | 1.75 (1.62, 1.88) | <.001 | 1.58 (1.46-1.72) | <.001 |
| 2 | 8991 (0.19) | 1826 (0.2) | 3.31 (3.08, 3.55) | <.001 | 2.94 (2.7-3.19) | <.001 |
| 3 | 2119 (0.05) | 775 (0.37) | 7.48 (6.76, 8.28) | <.001 | 5.24 (4.42-6.2) | <.001 |
| UO-Average | | | | | | |
| 0 | 16959 (0.37) | 1044 (0.06) | 1 [Reference] | NA | 1 [Reference] | NA |
| 1 | 9204 (0.2) | 924 (0.1) | 1.7 (1.55, 1.87) | <.001 | 1.48 (1.34-1.63) | <.001 |
| 2 | 15255 (0.33) | 2111 (0.14) | 2.45 (2.27, 2.65) | <.001 | 2.11 (1.93-2.31) | <.001 |
| 3 | 4926 (0.11) | 1573 (0.32) | 7.15 (6.56, 7.80) | <.001 | 5.59 (4.92-6.36) | <.001 |
| Block summation | | | | | | |
| 0 | 18927 (0.41) | 1231 (0.07) | 1 [Reference] | NA | 1 [Reference] | NA |
| 1 | 8248 (0.18) | 825 (0.1) | 1.6 (1.46, 1.75) | <.001 | 1.54 (1.4-1.69) | <.001 |
| 2 | 14830 (0.32) | 2143 (0.14) | 2.43 (2.26, 2.61) | <.001 | 2.38 (2.2-2.57) | <.001 |
| 3 | 4110 (0.09) | 1435 (0.35) | 7.71 (7.07, 8.41) | <.001 | 8.11 (7.29-9.03) | <.001 |
| **AUMCdb** | | | | | | |
| UO-Consecutive | | | | | | |
| 0 | 10235 (0.69) | 605 (0.06) | 1 [Reference] | NA | 1 [Reference] | NA |
| 1 | 2456 (0.16) | 384 (0.16) | 2.95 (2.57, 3.38) | <.001 | 2.91 (2.53-3.36) | <.001 |
| 2 | 1589 (0.11) | 428 (0.27) | 5.87 (5.11, 6.73) | <.001 | 5.16 (4.42-6.03) | <.001 |
| 3 | 643 (0.04) | 312 (0.49) | 15 (12.6, 17.9) | <.001 | 13.59 (10.49-17.62) | <.001 |
| UO-Average | | | | | | |
| 0 | 7494 (0.5) | 363 (0.05) | 1 [Reference] | NA | 1 [Reference] | NA |
| 1 | 2509 (0.17) | 278 (0.11) | 2.45 (2.08, 2.88) | <.001 | 2.34 (1.98-2.77) | <.001 |
| 2 | 3741 (0.25) | 570 (0.15) | 3.53 (3.08, 4.06) | <.001 | 3.34 (2.88-3.88) | <.001 |
| 3 | 1179 (0.08) | 518 (0.44) | 15.39 (13.2, 18.0) | <.001 | 12.74 (10.11-16.06) | <.001 |
| *^1^*Model include age, weight, gender and diagnosis at admission | | | | | | |

Next is the Kaplan-Meier (KM) survival plot for 30-day mortality, demonstrating higher survival differentiation for UOcons staging.


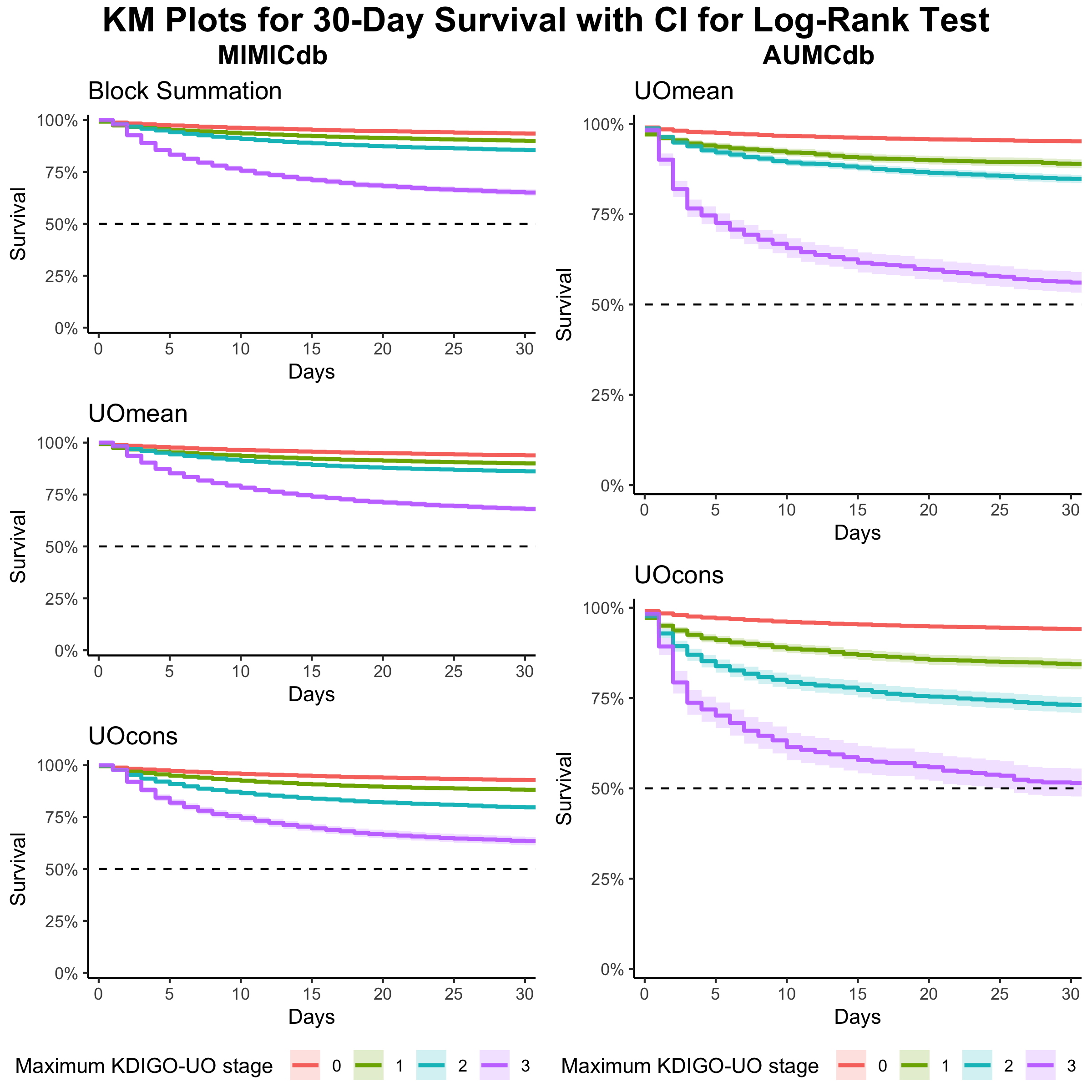

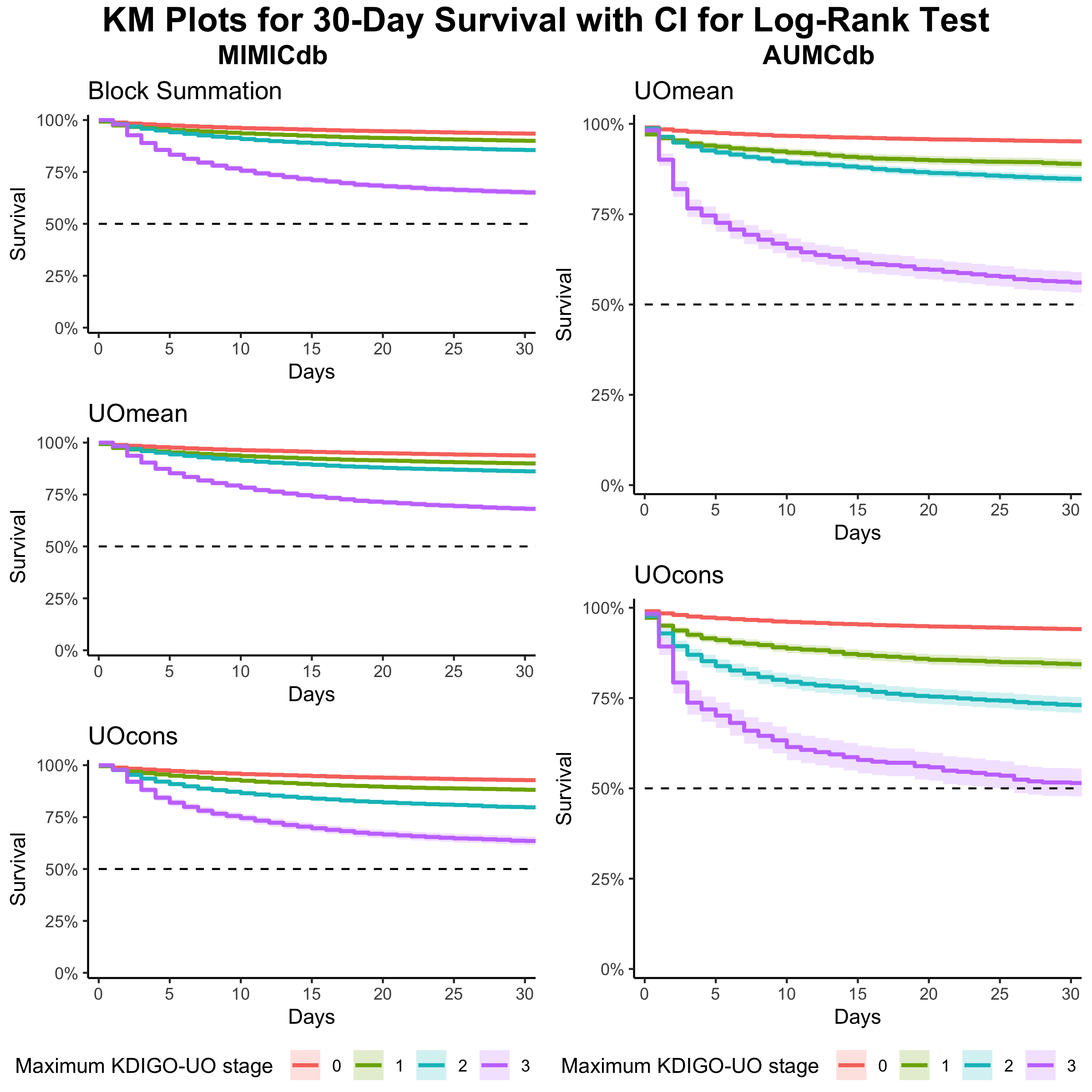


Lastly, KDIGO-UO interpretations were evaluated using model fit. To assess the impact of each KDIGO-UO interpretation on the association with 7-day mortality, a multivariate model was constructed with two variables: (1) Maximal KDIGO-UO staging within the first 72 hours of admission; and (2) Whether the initial calculated stage was positive. The second variable addresses the uncertainty of AKI onset and staging when diagnosed upon admission. The UOcons model demonstrated superior fit, while the block summation model had the poorest fit.

| **Model Comparison for the Association of 7-day Mortality with Different Interpretation of KDIGO-UO Criteria Using Bayesian Information Criterion (BIC)** | | |
| --- | --- | --- |
| Model | df | BIC |
| MIMICdb | | |
| model_block_summation | 3 | 20948.367 |
| model_UOmean | 3 | 20837.523 |
| model_UOcons | 3 | 20802.085 |
| AUMCdb | | |
| model_UOmean | 3 | 6926.717 |
| model_UOcons | 3 | 6903.629 |
| Models include both maximal staging within the first 72 hours of ICU admission and whether the first calculated stage was positive ('prevalence at admission'). | | |

# Supplementary Methods 2. Sample Size and Power Analysis

Minimal detectable differences (MDD) were assessed for the outcomes of in-hospital mortality, need for renal replacement therapy, and incidence of AKI in the first 72 hours of hospitalization. The MDDs were calculated for a series of different proportions of incidence. For the first two outcomes, *MDD-1* was calculated based on a total sample size of 85,378 eligible ICU admissions (67,642 in MIMICdb and 17,736 in AUMCdb). For the incidence of AKI, *MDD-2* was calculated based on a sample size of 61,346 eligible first ICU stays of each patient (46,423 in MIMICdb and 14,923 in AUMCdb). The analysis accounted for a power of 0.8 and an alpha of 0.05.

| Incidence Rate | MDD-1 | MDD-2 |
| --- | --- | --- |
| 0.1 | 0.3% | 0.3% |
| 0.2 | 0.4% | 0.5% |
| 0.3 | 0.4% | 0.5% |
| 0.4 | 0.5% | 0.6% |
| 0.5 | 0.5% | 0.6% |
| MDD-1: Minimal detectable difference between the MIMICdb and the AUMCdb in the incidence of either in-hospital mortality or renal replacment therapy; MDD-2: Minimal detectable difference between the MIMICdb and the AUMCdb in the incidence of AKI in the first day of ICU admission. | | |

# Supplementary Figure 1. Frequency of Urine Output Charting


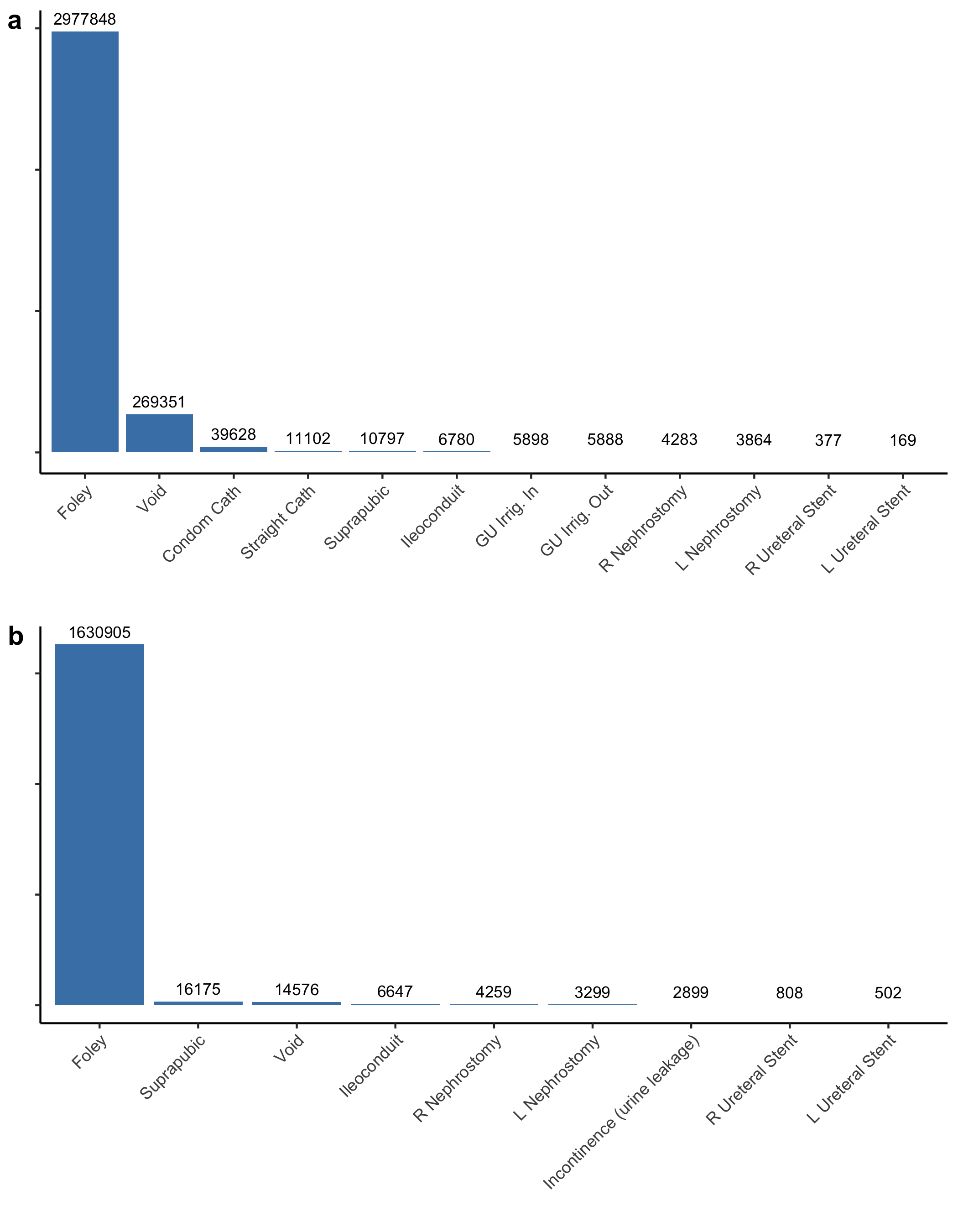


Frequency of urine output charting by the source before exclusions. (a) Among the 3,335,985 distinct urine output records in the MIMICdb; (b) Among the 1,573,533 distinct records in the AUMCdb. GU Irrig: Genitourinary Irrigation; L: Left; R: Right.

**Supplemental Appendix 4.** Age and Weight

Descriptive statistics and histograms for age (years):

| N | Mean | SD | 5th | 10th | 25th | 50th | 75th | 95th | Min | Max |
| --- | --- | --- | --- | --- | --- | --- | --- | --- | --- | --- |
| MIMICdb | | | | | | | | | | |
| 67642 | 64.79 | 16.79 | 32 | 41 | 55 | 66 | 77 | 89 | 18 | 102 |
| AUMCdb | | | | | | | | | | |
| 17736 | 63.36 | 15.22 | 29 | 45 | 55 | 65 | 75 | 85 | 29 | 85 |


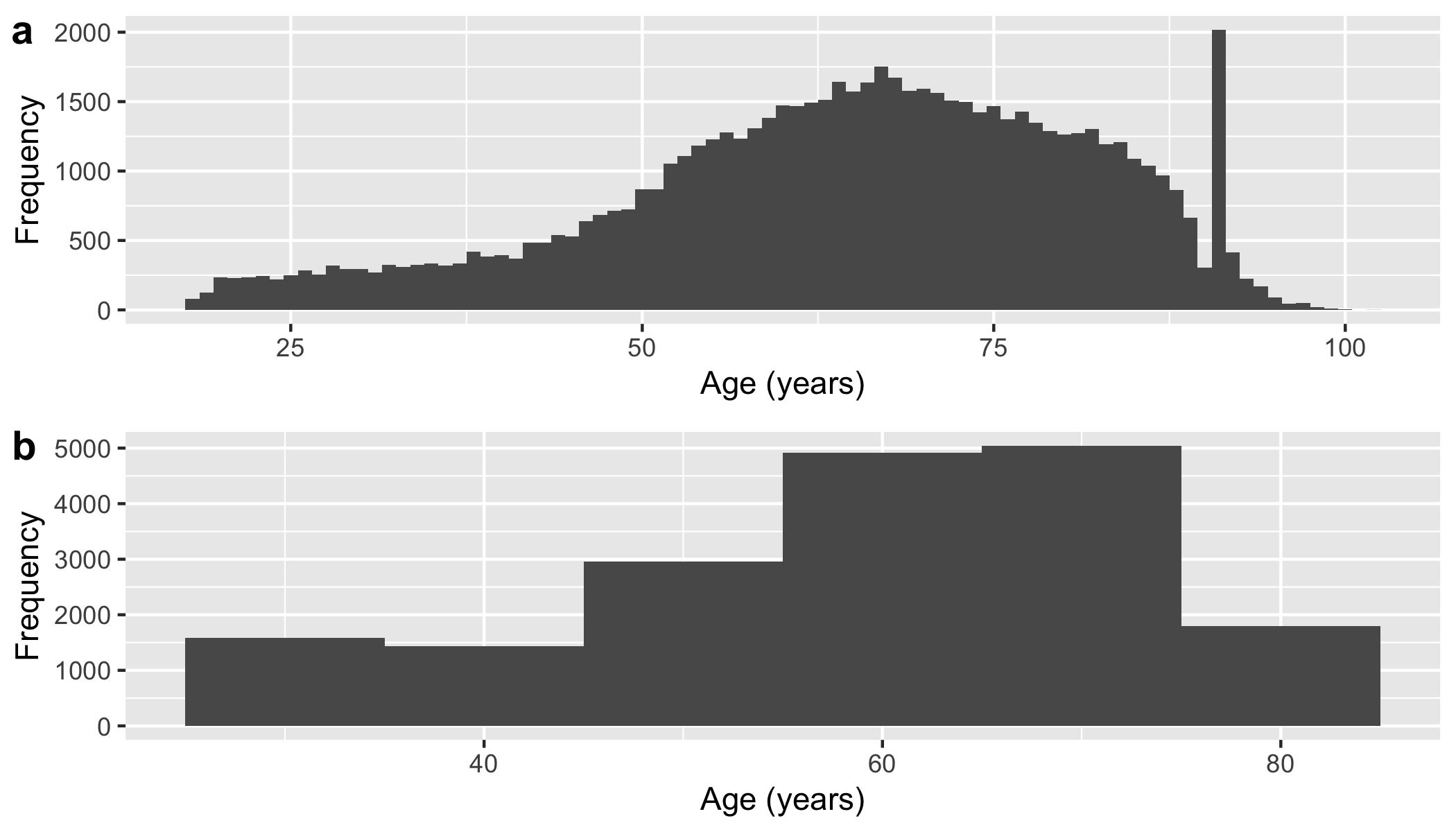


Histograms for age (years). Panel a plotted for MIMICdb; Panel b plotted for AUMCdb. For the N count, see the table above.

Descriptive statistics and histograms for weight (kg):

| N | Mean | SD | 5th | 10th | 25th | 50th | 75th | 95th | Min | Max |
| --- | --- | --- | --- | --- | --- | --- | --- | --- | --- | --- |
| MIMICdb | | | | | | | | | | |
| 65751 | 81.44 | 34.35 | 50 | 55.7 | 65.6 | 78.1 | 93 | 122 | 1 | 5864 |
| AUMCdb | | | | | | | | | | |
| 17143 | 80.54 | 14.52 | 55 | 65.0 | 75.0 | 75.0 | 85 | 105 | 55 | 115 |


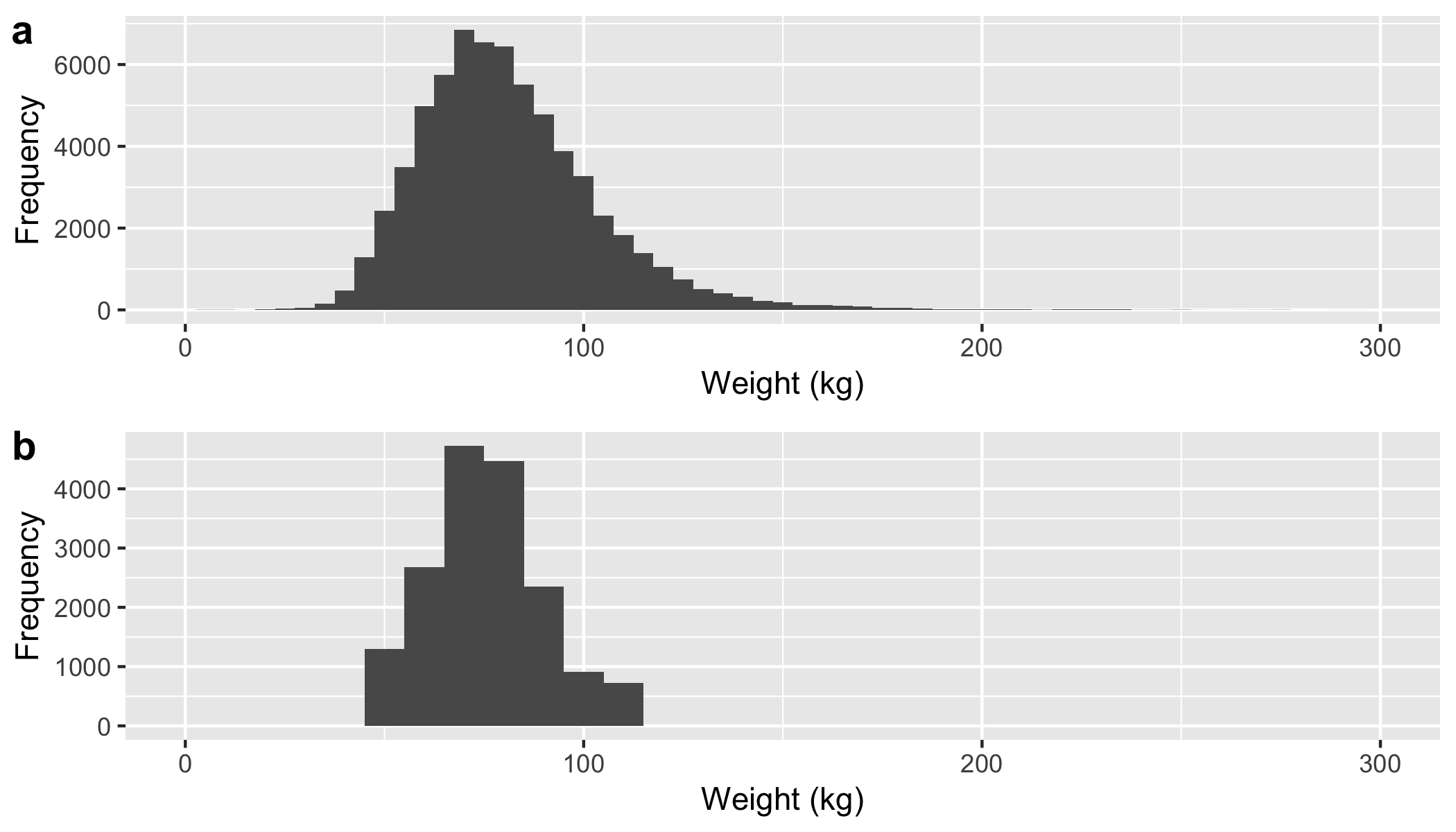


Histograms for weight (kg). Panel a plotted for MIMICdb; Panel b plotted for AUMCdb. For the N count, see the table above.

# Supplementary Table 2. Durations of Collection for All Volume Measurements

| Source | N | Mean | SD | 5th | 10th | 25th | 50th | 75th | 95th | Min | Max |
| --- | --- | --- | --- | --- | --- | --- | --- | --- | --- | --- | --- |
| MIMICdb | | | | | | | | | | | |
| Foley | 2851891 | 83 | 97 | 60 | 60 | 60 | 60 | 105 | 180 | 1 | 43235 |
| Void | 248862 | 232 | 375 | 60 | 60 | 119 | 180 | 287 | 585 | 1 | 51846 |
| Condom Cath | 37890 | 222 | 304 | 60 | 60 | 90 | 131 | 240 | 600 | 1 | 8904 |
| Straight Cath | 10207 | 732 | 1486 | 60 | 122 | 300 | 409 | 617 | 2547 | 1 | 35375 |
| Suprapubic | 9672 | 115 | 131 | 60 | 60 | 60 | 60 | 120 | 240 | 1 | 6616 |
| Ileoconduit | 6022 | 129 | 213 | 60 | 60 | 60 | 68 | 120 | 300 | 1 | 8040 |
| R Nephrostomy | 3465 | 223 | 243 | 60 | 60 | 120 | 180 | 240 | 635 | 2 | 7740 |
| L Nephrostomy | 3206 | 251 | 298 | 60 | 60 | 120 | 180 | 300 | 660 | 2 | 7560 |
| AUMCdb | | | | | | | | | | | |
| Foley | 1458673 | 86 | 87 | 60 | 60 | 60 | 60 | 120 | 120 | 1 | 26940 |
| Suprapubic | 11994 | 89 | 49 | 60 | 60 | 60 | 60 | 120 | 180 | 1 | 1080 |
| Void | 8284 | 158 | 383 | 60 | 60 | 60 | 120 | 180 | 420 | 1 | 21420 |
| Ileoconduit | 4749 | 129 | 794 | 60 | 60 | 60 | 120 | 120 | 228 | 1 | 35940 |
| R Nephrostomy | 3423 | 127 | 326 | 60 | 60 | 60 | 120 | 120 | 300 | 1 | 9720 |
| L Nephrostomy | 2404 | 139 | 361 | 60 | 60 | 60 | 120 | 120 | 300 | 1 | 9720 |

# Supplementary Figure 2. The Proportion of 'Zero Value' UO Measurements


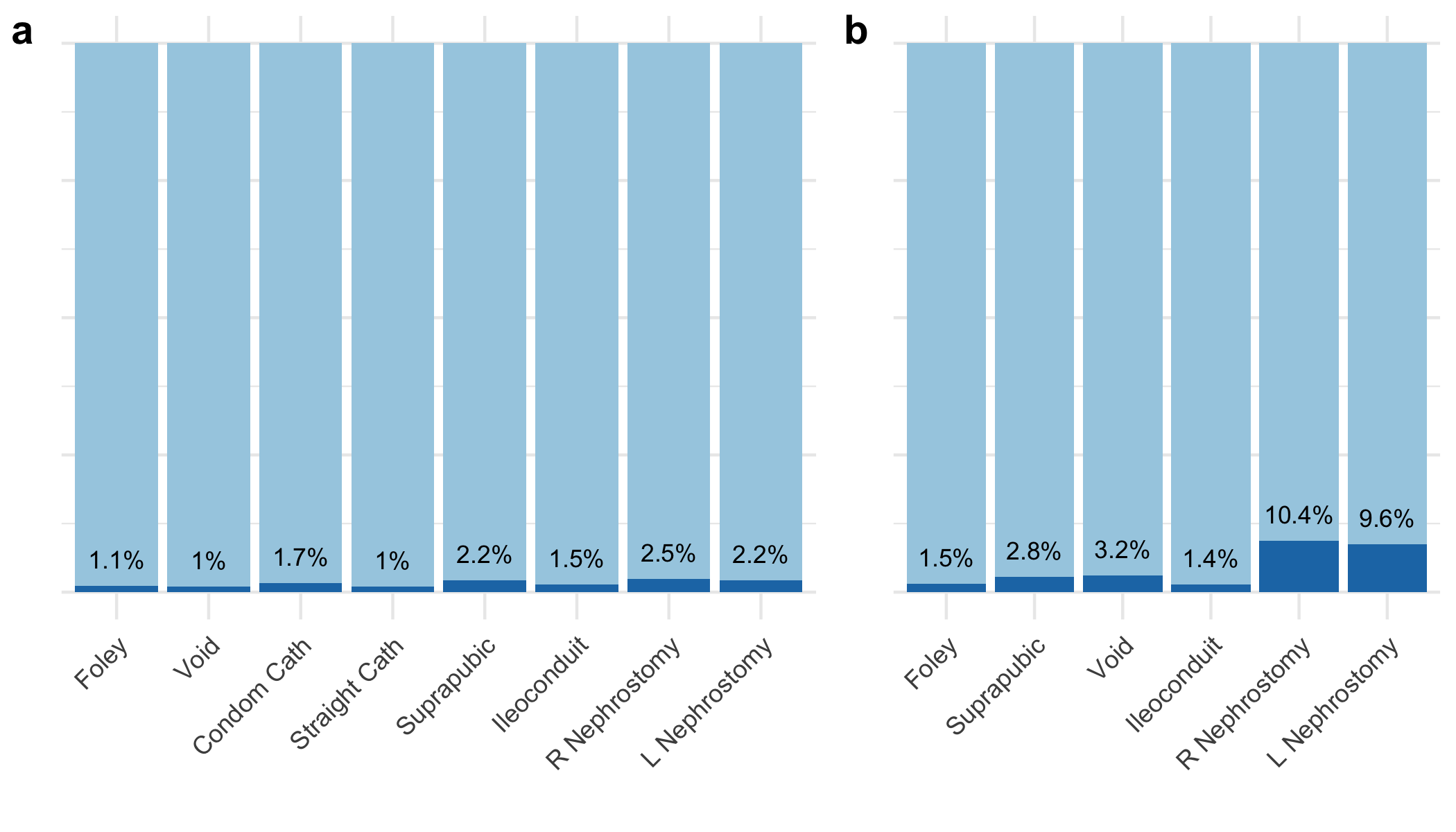


Proportion of zero volume charting. (a) Plotted for the MIMICdb; (b) Plotted for the AUMCdb. L: Left; R: Right.

# Supplementary Table 3. Durations of Collection for Zero-Volume Measurements

| Source | N | Mean | SD | 5th | 10th | 25th | 50th | 75th | 95th | Min | Max |
| --- | --- | --- | --- | --- | --- | --- | --- | --- | --- | --- | --- |
| MIMICdb | | | | | | | | | | | |
| Foley | 31574 | 103 | 232 | 45 | 60 | 60 | 60 | 104 | 240 | 1 | 15007 |
| Void | 2609 | 412 | 1078 | 39 | 60 | 60 | 180 | 300 | 1440 | 1 | 18747 |
| Condom Cath | 651 | 194 | 388 | 51 | 60 | 60 | 120 | 240 | 516 | 1 | 5952 |
| Suprapubic | 213 | 222 | 230 | 33 | 60 | 60 | 210 | 240 | 528 | 1 | 2189 |
| Straight Cath | 101 | 1013 | 1859 | 60 | 120 | 180 | 303 | 858 | 3508 | 13 | 14427 |
| Ileoconduit | 89 | 216 | 292 | 60 | 60 | 60 | 120 | 240 | 699 | 15 | 1680 |
| R Nephrostomy | 85 | 275 | 873 | 60 | 60 | 60 | 120 | 180 | 516 | 13 | 7740 |
| L Nephrostomy | 70 | 258 | 892 | 60 | 60 | 60 | 120 | 220 | 360 | 60 | 7560 |
| AUMCdb | | | | | | | | | | | |
| Foley | 22280 | 114 | 245 | 60 | 60 | 60 | 60 | 120 | 300 | 1 | 20868 |
| R Nephrostomy | 355 | 163 | 527 | 60 | 60 | 60 | 120 | 120 | 360 | 1 | 9720 |
| Suprapubic | 337 | 106 | 87 | 60 | 60 | 60 | 60 | 120 | 240 | 1 | 600 |
| Void | 265 | 257 | 475 | 60 | 60 | 60 | 120 | 240 | 996 | 1 | 4560 |
| L Nephrostomy | 231 | 184 | 373 | 60 | 60 | 60 | 120 | 120 | 540 | 59 | 5040 |
| Ileoconduit | 65 | 587 | 3425 | 60 | 60 | 60 | 60 | 120 | 456 | 1 | 27480 |

# Supplementary Appendix 4. Rates vs Durations of Collection

The figure below illustrates the relationship between the urine output (UO) rate and the time interval between UO measurements. As the UO rate increases within the displayed range, the time interval converges toward a median of 60 minutes. Conversely, as the UO rate decreases, the time interval lengthens, with a marked increase observed at rates below 20 mL/hr.


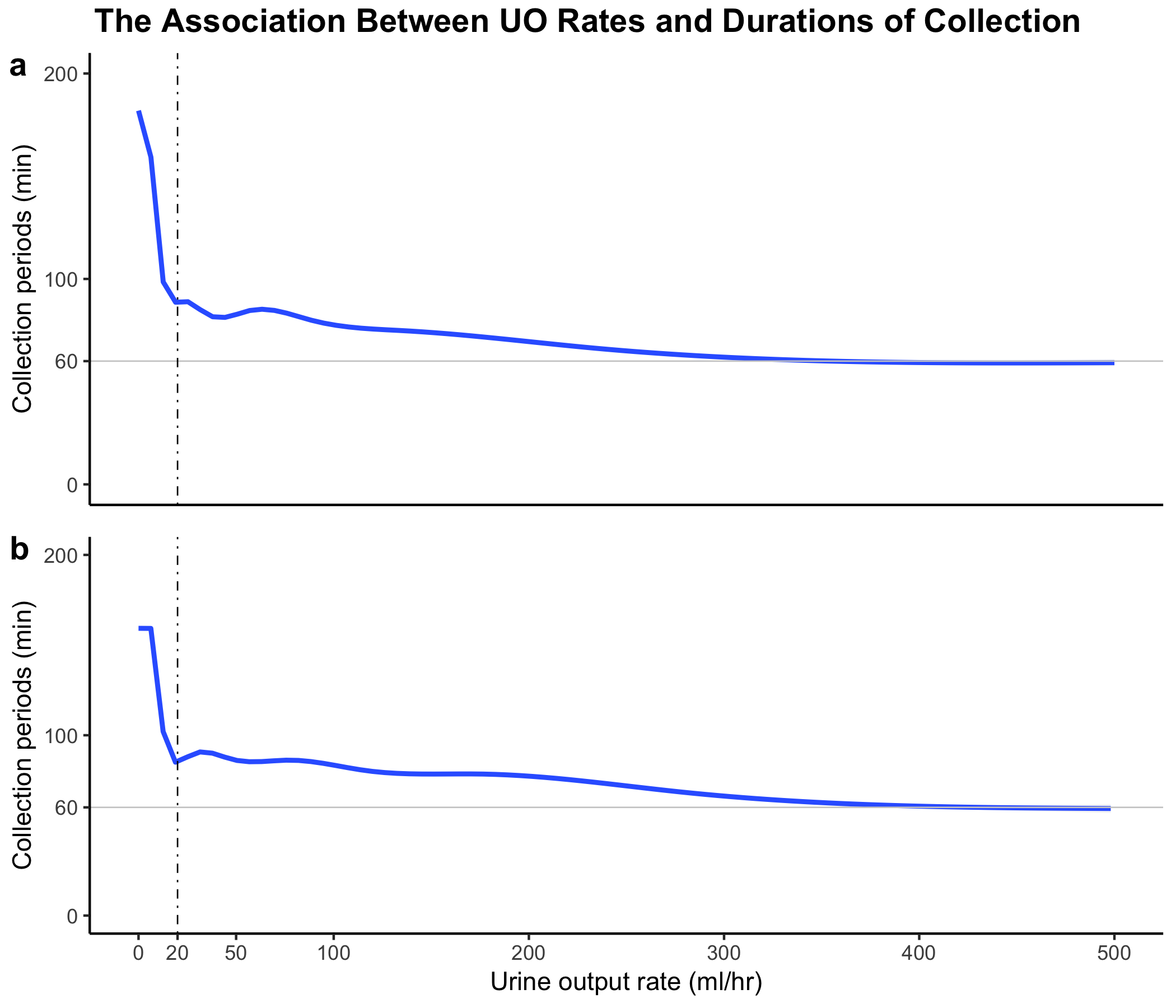


This figure shows the association between urine output rates and durations of collection for Foley catheters. The blue line represents the smoothed conditional mean of urine output rates, and the standard error is hidden under the line due to its smaller size. The solid grey is set to 60 minutes of collection time, while the dot-dashed line indicates an artificially set threshold due to a sharp increase in urine output rates. Approximately 10% of all measurements fall below this threshold. (a) Plotted for the MIMICdb; (b) Plotted for the AUMCdb.

For the MIMICdb, the quantile analysis below reveals no significant correlation between UO rates and time intervals between percentiles 0.1-0.7. This is expected, as the UO rate is calculated by dividing urine volume by the time interval. Therefore, normal physiological variations in the UO rate would not directly influence time intervals in this range. However, a trend emerges in the upper percentiles (0.8-0.9): significantly increasing negative correlations appear between UO rates and time intervals. Notably, extremely long intervals coincide with low UO rates, and conversely, higher UO rates within these percentiles are associated with shorter intervals between measurements.

As for the AUCMdb, the figure demonstrates a slightly different pattern. Within this population, only the 0.6th percentile exhibits a significant correlation with low UO rates, unlike the broader trend observed in MIMICdb for percentiles 0.7-0.9.


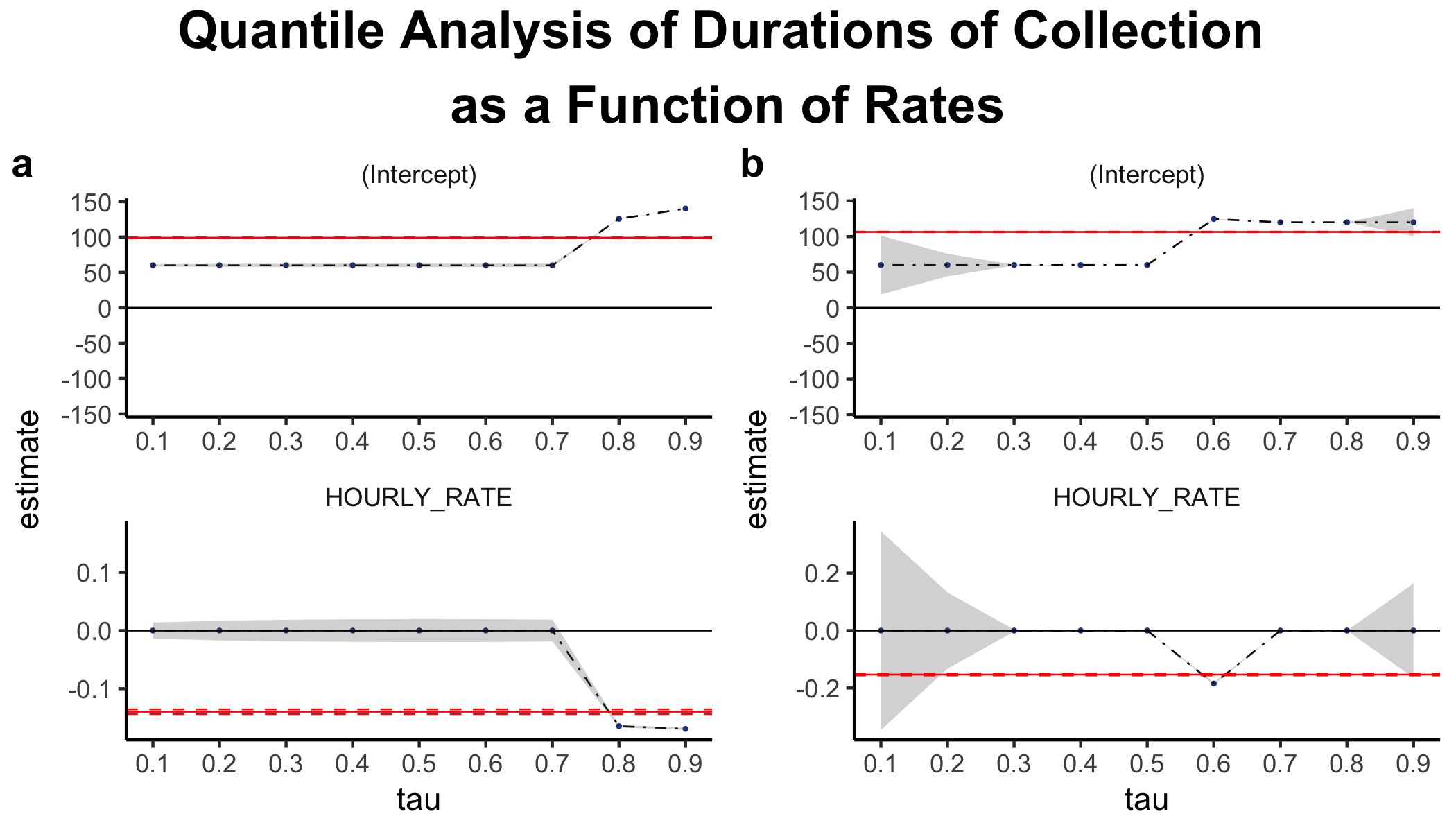


The figure shows the slope and intercept of the estimated linear quantile regression for the percentiles of the collection period as a function of tau, with urine output rates as the only variable. The estimate is shown with blue dots and a confidence interval in the gray area. The estimate and confidence interval for a linear model are also shown in red. The analysis was performed on a sample of the most recent 500,000 Foley catheter measurements. (a) Plotted for the MIMICdb; (b) Plotted for the AUMCdb.

The next figure illustrates key differences between the two datasets in their temporal recording patterns. While the number of points is the same in both panels, Panel a shows more points than Panel b. This disparity reflects the recording bias in AUMCdb towards round hours, leading to superimposed data points at these specific time points. As a result, many points in panel a “fall” on top of each other at the horizontal lines of the round hours.

In Panel a, in addition to the horizontal lines of the round hours, distinct trend lines, displaying negative exponential curves with regular intervals, form above the 60-minute line. Below this line, symmetrical recurring patterns of negative and positive exponential curves cluster around a vertical point. Notably, these trends are also present in Panel b, albeit less pronounced due to the aforementioned recording bias.

For durations of collection exceeding one hour, the data exhibits volume-dependent clustering manifested by pronounced curves representing multiples of 50 ml volume records above the 60-minute line. These also exist for multiples of 25 ml but are less pronounced. For example, in Panel a, all points representing 300 ml volumes coalesce into a curve intersecting the (300ml, 60min) point and extending to the intersection of point (150ml, 120min).

Similarly, the grouping of points below the 60-minute durations of collection also reveals volume-dependency, characterized by discrete jumps, most often of 25ml, and no less than 5ml, albeit less frequently. This pattern arises from the limitations of dividing discrete minute recordings by 60, impacting factorability. As an example, the vertical cluster around a urine output rate of 250 ml/hour in Panel B results from dividing an integer multiple of a 25 ml volume measurement by an integer multiple of a 6-minute interval.

The findings described above provide insight into the results of the quantile analysis. Notably, in the figure below, regression lines for percentiles aligned with round-hour durations of collection (tau 0.1-0.7 in Panel a and tau 0.1-0.5, 0.7-0.9 in Panel b) are horizontal, indicating no correlation with urine output rate. In contrast, the regression lines of percentiles that fall on non-round-hour durations of collection demonstrate a negative association with urine output rate (tau 0.8-0.9 in panel a and tau 0.6 in panel b). This is likely due to frequent urine recording at round hours obscuring the relationship between low urine output and longer urine durations of collection. In the AUMCdb, which is characterized by frequent hourly urine recording, this relationship was more difficult to detect. These results are also congruent with the figure above.

**Scatterplot and Quantile Regression Fit of Durations of Collection as a Function of Rates**


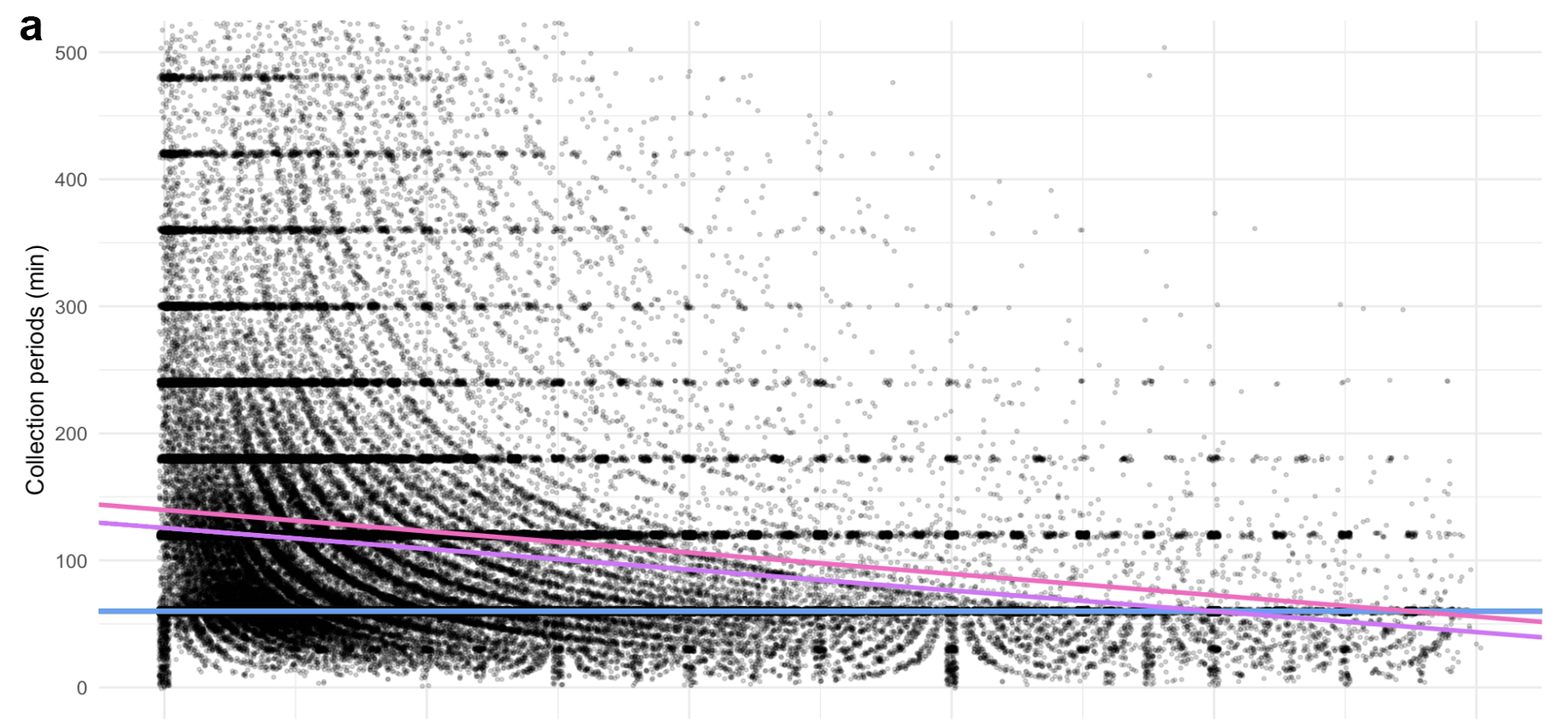


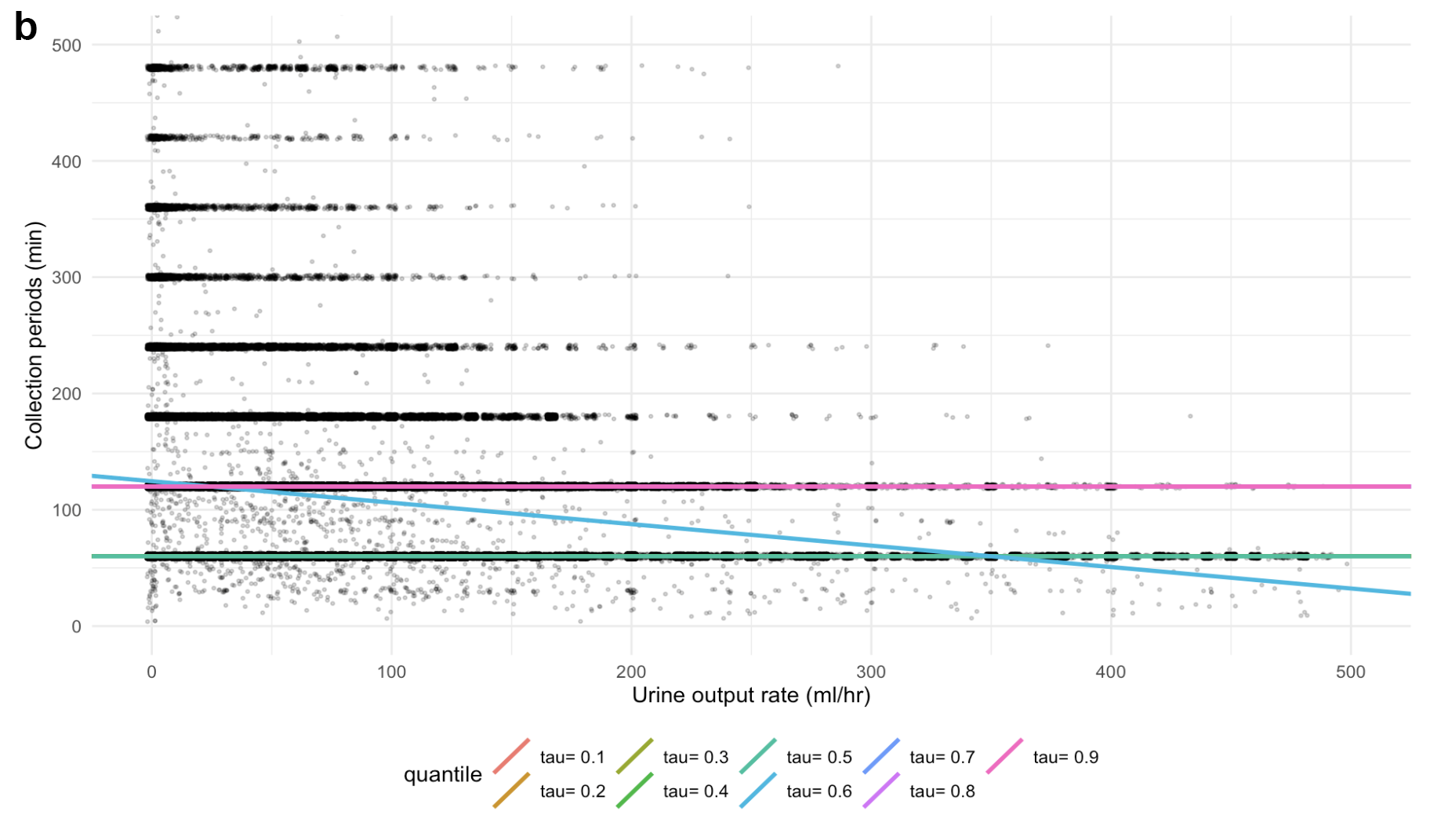


This figure shows a scatterplot of urine output vs. collection time with superimposed quantile regression lines representing various percentiles of the UO durations of collection (tau = 0.1–0.9). To improve readability with densely packed data, 20% transparency and jitter of 2 units were applied in both positive and negative directions, horizontally and vertically. Analysis based on the most recent 500,000 Foley catheter measurements. (a) Plotted for the MIMICdb; (b) Plotted for the AUMCdb.

# Supplementary Figure 3. Flowchart of Urine Data Selection and Adjustment Process in AUMCdb

# Supplementary Table 4. Comparison of Hourly-Adjustment vs Hourly Summation

| **Cut-off (ml)** | **Proportion of Agreement** | **Proportion of Disagreement** |
| --- | --- | --- |
| MIMICdb | | |
| <10 | 45.4% | 54.6% |
| <50 | 66.6% | 33.4% |
| <100 | 84.2% | 15.8% |
| <150 | 91.7% | 8.3% |
| <200 | 95.2% | 4.8% |
| AUMCdb | | |
| <10 | 29.5% | 70.5% |
| <50 | 60.1% | 39.9% |
| <100 | 82.2% | 17.8% |
| <150 | 92% | 8% |
| <200 | 96.1% | 3.9% |
| The table demonstrates the significance of hourly adjustment for accuracy by presenting the variance between the adjusted values and the simple hourly summation. Cut-off values are based on the absolute difference between the hourly-adjusted UO and a simple hourly summation of UO. Measurements charted on the hour were included with the previous time interval. | | |
